# Personal Decision-Making Criteria Related to Seasonal and Pandemic A(H1N1) Influenza-Vaccination Acceptance among French Healthcare Workers

**DOI:** 10.1371/journal.pone.0038646

**Published:** 2012-07-27

**Authors:** Lila Bouadma, François Barbier, Lucie Biard, Marina Esposito-Farèse, Bertrand Le Corre, Annick Macrez, Laurence Salomon, Christine Bonnal, Caroline Zanker, Christophe Najem, Bruno Mourvillier, Jean Christophe Lucet, Bernard Régnier, Michel Wolff, Florence Tubach, for the INFLUENCE-A Study Group

**Affiliations:** 1 Réanimation Médicale et des Maladies Infectieuses, Hôpital Bichat–Claude-Bernard Hôpitaux Universitaires Paris Nord–Val de Seine, Assistance Publique–Hôpitaux de Paris, Paris, France; 2 EA 3964, Université Paris Diderot, Paris, France; 3 Département d'Epidémiologie Biostatistique et Recherche Clinique, Hôpital Bichat–Claude-Bernard Hôpitaux Universitaires Paris Nord–Val de Seine, Assistance Publique–Hôpitaux de Paris, Paris, France; 4 Direction de la Qualité et Gestion des Risques, Hôpital Bichat–Claude-Bernard Hôpitaux Universitaires Paris Nord–Val de Seine, Assistance Publique–Hôpitaux de Paris, Paris, France; 5 Département de Santé Publique, Hôpital Louis-Mourier Hôpitaux Universitaires Paris Nord–Val de Seine, Assistance Publique–Hôpitaux de Paris, Colombes, France; 6 Unité d'Hygiène et de Lutte contre l'Infection Nosocomiale, Hôpital Bretonneau Hôpitaux Universitaires Paris Nord–Val de Seine, Assistance Publique–Hôpitaux de Paris, Paris, France; 7 Urgences Médicales/Urgences Chirurgicales, Hôpital Beaujon Hôpitaux Universitaires Paris Nord–Val de Seine, Assistance Publique–Hôpitaux de Paris, Clichy, France; 8 Service de Pharmacie/Hygiène Hôpital Charles-Richet Hôpitaux Universitaires Paris Nord–Val de Seine, Assistance Publique–Hôpitaux de Paris, Villiers-le-Bel, France; 9 Unité d'Hygiène et de Lutte contre l'Infection Nosocomiale, Hôpital Bichat–Claude-Bernard Hôpitaux Universitaires Paris Nord–Val de Seine, Assistance Publique–Hôpitaux de Paris, Paris, France; 10 Université Paris Diderot-Sorbonne, Paris, France; 11 CIE 801, Institut National de la Santé et de la Recherché Médicale, Paris, France; University of Hyderabad, India

## Abstract

**Background:**

Influenza-vaccination rates among healthcare workers (HCW) remain low worldwide, even during the 2009 A(H1N1) pandemic. In France, this vaccination is free but administered on a voluntary basis. We investigated the factors influencing HCW influenza vaccination.

**Methods:**

In June–July 2010, HCW from wards of five French hospitals completed a cross-sectional survey. A multifaceted campaign aimed at improving vaccination coverage in this hospital group was conducted before and during the 2009 pandemic. Using an anonymous self-administered questionnaire, we assessed the relationships between seasonal (SIV) and pandemic (PIV) influenza vaccinations, and sociodemographic and professional characteristics, previous and current vaccination statuses, and 33 statements investigating 10 sociocognitive domains. The sociocognitive domains describing HCWs' SIV and PIV profiles were analyzed using the classification-and-regression–tree method.

**Results:**

Of the HCWs responding to our survey, 1480 were paramedical and 401 were medical with 2009 vaccination rates of 30% and 58% for SIV and 21% and 71% for PIV, respectively (p<0.0001 for both SIV and PIV vaccinations). Older age, prior SIV, working in emergency departments or intensive care units, being a medical HCW and the hospital they worked in were associated with both vaccinations; while work shift was associated only with PIV. Sociocognitive domains associated with both vaccinations were self-perception of benefits and health motivation for all HCW. For medical HCW, being a role model was an additional domain associated with SIV and PIV.

**Conclusions:**

Both vaccination rates remained low. Vaccination mainly depended on self-determined factors and for medical HCW, being a role model.

## Introduction

The 2009 A(H1N1) influenza was declared a pandemic on 11 June 2009 [1] and, on 13 July the World Health Organization (WHO) defined healthcare workers (HCW) as the priority target for A(H1N1) vaccination campaigns [2]. The Centers for Disease Control and Prevention (CDC) [3] and other major public health institutions [4], have recommended annual seasonal influenza vaccination (SIV) for all HCW for many years. Indeed, vaccination reduces HCW absenteeism and may preserve healthcare services during flu outbreaks [5]. Moreover, infected HCW can be vectors for nosocomial influenza [6]. Despite recommendations and vaccination campaigns, influenza vaccination rates among HCW remained constantly below 60% in the United States [7] and only 13%–48% in European countries [8]. Although numerous strategies have been developed to improve influenza-vaccination acceptance among HCW, they resulted in minimal changes of vaccination rates [9], while mandatory vaccination raises ethical concerns [10]. Furthermore, during the 2009 pandemic alert and its intense media coverage, vaccination rates remained low [11], highlighting the need for better understanding of HCWs' decision-making during the alert to plan future campaigns.

Encouraging HCW influenza vaccination is a complex issue. During the past 50 years, the assumption that an individual's perception strongly influenced his/her behavior gave rise to sociocognitive models of human behavior [12]. Previous findings suggested several reasons that might lead to HCW vaccination refusal [13]. However, to date, individual sociocognitive factors affecting influenza-vaccination acceptance have not been studied concurrently among HCW.

Our study aimed to investigate, in an unprecedented pandemic context, the sociodemographic and work-related factors associated with SIV and pandemic A(H1N1) influenza vaccination (PIV) among HCW, and to identify sociocognitive profiles reflecting decision-making concerning both.

**Figure 1:**
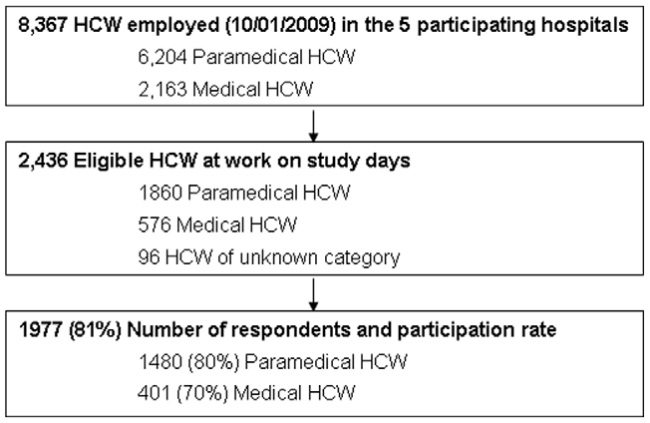
Study flow chart. HCW  =  healthcare workers.

**Table 1:** Sociodemographic and professional characteristics of the 1,881 study respondents.

Characteristics, n(%)	Paramedical HCW (N = 1,480)	Medical HCW (N = 401)
Age (year), mean (SD)	36.2 (10.5)	33.7 (10.6)
Female	1,225/1,475 (83.1)	230/400 (57.5)
Chronic disease	109/1,446 (7.5)	18/398 (4.5)
Living alone	211/1,407 (15.0)	78/395 (19.7)
Children in the household	364/1,147 (31.7)	86/294 (29.3)
Pregnant	41/1,092 (3.8)	14/287 (4.9)
Type of ward		
*ICU/ED*	314/1,418 (22.1)	116/396 (29.3)
Medical* or Surgical	783/1,418 (55.2)	257/396 (64.9)
*Geriatric*	321/1,418 (22.6)	23/396 (5.8)
SIV during the 2006, 2007 and 2008 campaigns		
*Never*	808/1,431 (56.5)	121/395 (30.6)
*Once*	269/1,431 (18.8)	77/395 (19.5)
*Twice*	127/1,431 (8.9)	61/395 (15.4)
*Always*	227/1,431 (15.9)	136/395 (34.4)

HCW = healthcare workers. ICU/ED = intensive care unit/emergency department. SIV = seasonal influenza vaccination. *Including obstetrics and pediatrics.

## Methods

Additional methodologic details are given as supportive informations (Methods S1).

### Ethics Statement

The Institutional Review Board (Comité d'Ethique du Groupe Hospitalo-Universitaire Paris-Nord, IRB no. 00006477) approved the study protocol and waived the need for written informed consent of the participants.

### Study characteristics

On a given day, 8,367 HCW in a five-hospital French university healthcare group located in Northern Paris and its suburbs participated in a cross-sectional study. This group has 2,622 beds with diversified and complementary activities (see Table S1). Bichat–Claude-Bernard Hospital, a tertiary-care center for infectious diseases and pandemic influenza, has 987 beds; the 472-bed Beaujon Hospital has mostly surgical activities; Charles-Richet (472 beds) and Bretonneau (205 beds) Hospitals are dedicated to geriatric care; Louis-Mourier Hospital has 486 beds, including acute care, rehabilitation and long-term–care units.

HCW are defined as medical (doctors, medical students and midwives) and paramedical (nurses, nurses' aides, physiotherapists and orderlies) based on the authorization to prescribe.

In 2009, this group conducted a multifaceted PIV and SIV campaign, combining education and encouragement, free and convenient vaccination, real-time feedback on vaccination rates to each department, involvement of all hospital leaders and administration support (see Methods S1).

Free SIV and PIV were offered to all HCW and were administered free-of-charge from 1 September to 20 October 2009, directly in the ward or the occupational medicine unit. Both vaccinations were subsequently available throughout the pandemic-influenza period.

The survey was conducted over two consecutive weeks, between 25 June and 8 July 2010, over 24 hours the same weekday in each hospital. All HCW working in inpatient wards the study day were eligible to participate, regardless of their shift (night or day). HCW not directly in charge of patients (secretaries and technical, laboratory and administrative staff) and those from outpatient clinics, operating rooms and radiology departments were not included. A study-dedicated monitor individually distributed anonymous self-administered questionnaires to all HCW present in each hospital over the defined 24-hour period, emphasizing the questionnaire's anonymous nature and encouraging HCW to complete it before placing it in an anonymous envelope that the monitors collected the same day.

**Figure 2:**
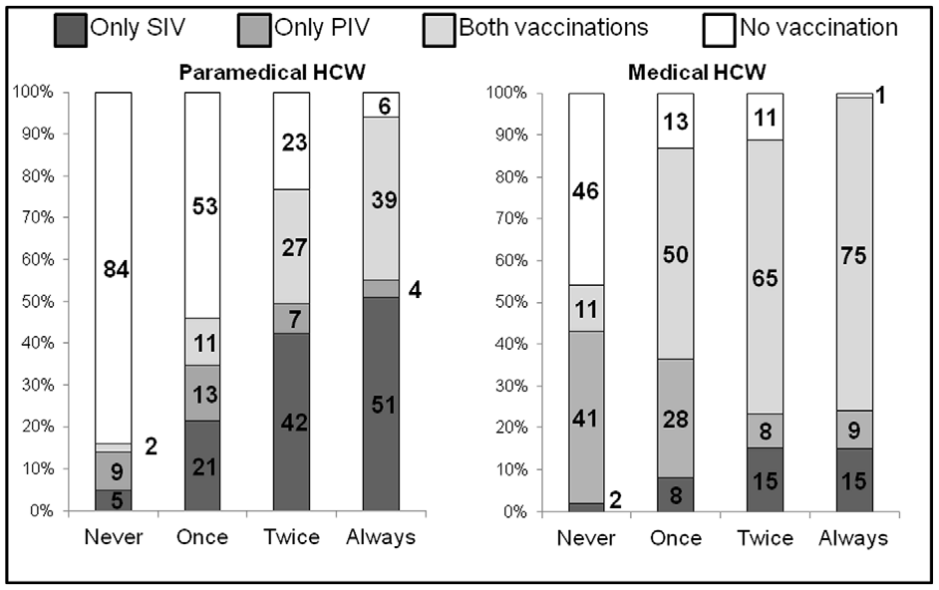
Influence of prior seasonal influenza vaccinations according to current vaccination status for paramedical and medical healthcare workers. SIV = seasonal influenza vaccination. PIV = pandemic influenza vaccination. HCW  =  healthcare workers.

### The questionnaire

Four investigators designed and validated the questionnaire (Appendix S1).

Part I of the questionnaire addresses demographic, personal, family and professional characteristics, history of influenza vaccination, and current SIV and PIV statuses.

Part II consists of 33 statements, reflecting sociocognitive theories applied to health behaviors, adapted to the context of influenza vaccination and addressing potential individual sociocognitive factors of influenza-vaccination acceptance (see Table S2) [13]–[18], grouped into 10 cognitive dimensions: 1) self-perception of susceptibility (self-opinion of the likelihood of disease acquisition and transmission, n = 5); 2) self-perception of seriousness (self-opinion of the seriousness of consequences if the disease is contracted, n = 5); 3) self-perception of benefits (self-opinion of the potential benefits of the recommended preventive health action to reduce the risk or seriousness of impact, n = 4); 4) self-perception of barriers (self-opinion of the barriers to accepting the recommended preventive health action, n = 5); 5) self-perception of own knowledge (self-opinion concerning own knowledge of the disease and the recommended preventive health action, n = 2); 6) self-perception of behavioral norm (self-opinion of how compliant colleagues are with the recommended preventive health action, n = 2); 7) self-perception of subjective norm (self-opinion of the expectations of others (whom I admire) on how I comply with the recommended preventive health action, n = 4); 8) beliefs (self-opinion of a false statement concerning the recommended preventive health action, n = 1); 9) health motivation (self-opinion of the likelihood of the recommended preventive health action to preserve health, n = 1); and 10) self-perception of external influences (self-opinion of impact of external influences on accepting the recommended preventive health action, n = 4).

According to the dimension, when pertinent, a statement referred to the HCW, his/her family circle or patients (see Table S2 for definitions and Appendix S1 for related questionnaire items). Another more global item assessing the perception of the risk-benefit balance was added: “I thought that the benefit of flu vaccination was greater than its related risks”. The 34 items were used twice in the final questionnaire, first to address SIV and then PIV. HCW had to rate their degree of agreement with each statement with a Likert five-point scale (from strongly agree to strongly disagree).

### Statistical analyses

The sample size was the number of HCW in the five hospitals who completed the questionnaire. The denominator of the participation rate (i.e., medical and paramedical HCW present on the study day) was obtained from each ward's administrative staff. Because response rates differed widely between medical and paramedical HCW, these categories were considered separately, thereby excluding from all the analyses the 96 HCW of unknown paramedical or medical status.

For each of the vaccination status SIV or PIV, factors associated were identified for the entire population by univariate analyses. Potential factors considered for both vaccination status were sex, age, prior SIV at least once during the three previous winters, work shift (day only, night only, or alternating day/night), living alone or with others, pregnancy, chronic disease (i.e., cardiac disease, cancer, history of stroke, chronic respiratory failure, diabetes mellitus, and/or immunosuppression), professional status (paramedical or medical HCW), and ward (intensive care unit (ICU) or emergency department (ED), acute medical (including obstetric and pediatric) or surgical, rehabilitation or long-term care, others or unknown). For multivaried analysis, logistic regressions were fitted for each vaccination status separately. Adjusting factors in the models were those significantly associated (P≤0.1) in univariate analyses or potentially important. For SIV the adjusting factors were pandemic vaccination, age, working hours, professional category, ward and center. For PIV the adjusting factors were seasonal vaccination, gender, age, working hours, professional category, ward, living alone, pregnancy and center. Final models were selected with stepwise procedures (backward, forward and both). All final models regardless of stepwise regression gave the same results.

To identify homogeneous profiles of paramedical and medical HCW in terms of the individual sociocognitive items, these factors were subjected to a decision-tree–classification method based on recursive partitioning analysis. The endpoint-of-interest was whether or not the HCW chose vaccination. The classification-and-regression trees (CART) were performed with the “rpart” package [19], which implements CART [20] ideas using R software v.2.12 [21].

**Table 2:** Factors associated with healthcare workers' vaccinations against seasonal and pandemic A(H1N1) influenza: results of multivariate analysis.

	Adjusted Odds ratio* [95% CI]	P Value
**Factors associated with SIV**		
Age, per-10 year increment	1.25 [1.07–1.47]	0.0057
SIV history during the past 3 previous winters		<0.0001
*Never*	1	
*Once*	6.0 [4.12–8.73]	
*Twice*	31.89 [19.67–51.69]	
*Always*	87.40 [52.90–144.42]	
Ward		0.0004
*ICU/ED*	1	
*Medical* * *or surgical*	0.48 [0.33–0.71]	
*Geriatric*	0.29 [0.09–0.89]	
Professional category		<0.0001
*Paramedical*	1	
*Medical*	2.10 [1.45–3.03]	
Hospital		0.042
*Beaujon*	1	
*Bichat*	0.87 [0.58–1.29]	
*Bretonneau*	2.83 [0.78–10.29]	
*Charles Richet*	1.51 [0.42–5.40]	
*Louis Mourier*	1.58 [0.98–2.56]	
**Factors associated with PIV**		
Age, per-10 year increment	1.25 [1.08–1.45]	0.009
SIV history during the past 3 previous winters		<0.0001
*Never*	1	
*Once*	2.75 [1.90–3.97]	
*Twice*	3.75 [2.40–5.85]	
*Always*	4.97 [3.44–7.18]	
Ward		<0.0001
*ICU/ED*	1	
Medical** or Surgical	0.54 [0.39–0.75]	
*Geriatric*	0.17 [0.05–0.62]	
Professional category		<0.0001
*Paramedical*	1	
*Medical*	7.71 [5.55–10.71]	
Hospital		<0.0001
*Beaujon*	1	
*Bichat*	2.73 [1.90–3.93]	
*Bretonneau*	4.22 [1.04–17.17]	
*Charles Richet*	1.28 [0.29–5.74]	
*Louis Mourier*	2.14 [1.37–3.33]	
Working hours		<0.0001
*Night shifts*	1	
*Day & day/night shifts*	2.65 [1.60–4.38]	
Living alone		0.066
*Yes*	1	
*No*	1.44 [0.97–2.12]	
Pregnancy		0.128
*No*	1	
*Yes*	0.49 [0.19–1.23]	

* For SIV the adjusting factors were pandemic vaccination, age, working hours, professional category, ward and center. For PIV the adjusting factors were seasonal vaccination, gender, age, working hours, professional category, ward, living alone, pregnancy and center. **Including obstetrics and pediatrics.

Two distinct partitionings were done: one focusing on SIV (S Questionnaire items) and the other on PIV status (A Questionnaire items). Separate analyses were performed for paramedical and medical HCW. Analyses were repeated separately for all HCW with prior SIV combined. Additional item 34 on the perceived benefit risk of the vaccination was not included, given that it summarizes different cognitive dimensions.

Associations between perceived vaccination benefit/risk and current vaccination status were evaluated separately with χ^2^ tests for paramedical and medical HCW.

## Results

### Study Participants

A total of 2,436 eligible paramedical and medical HCW (n = 1,860 and 576, respectively) were at work on the study days in the five participating hospitals. Among them, 1,977 HCW participated in the study (overall response rate, 81%), including 1,480 paramedical and 401 medical HCW (respective response rates, 80% and 70%, and 96 of unknown professional status excluded from the analyses, leaving 1,881 HCW for the study (Figure 1). Respondents' sociodemographic and professional characteristics are shown in Table 1, with distributions and response rates for each hospital and according to HCW category in Figure S1 and Table S3. Participation rates were similar for the 5 hospitals.

**Figure 3:**
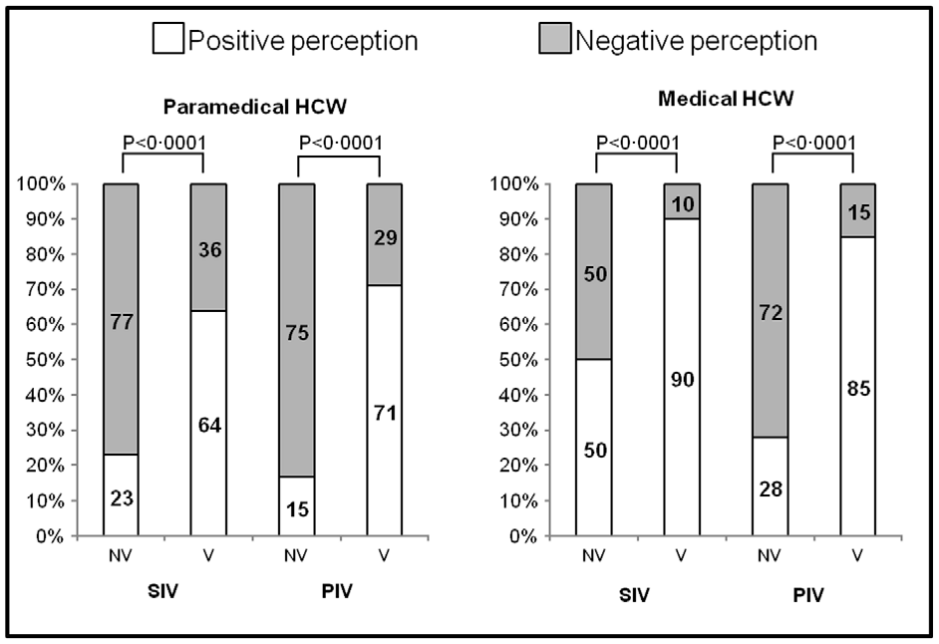
Perception of the benefit/risk ratio of vaccination, according to current vaccination status for paramedical and medical healthcare workers. NV = nonvaccinated. V = vaccinated. HCW = healthcare workers. A positive perception was defined as agreement (agree, or strongly agree) with the statement “I thought that the benefit of flu vaccination was greater than its related risks”.

Numbers of missing data per item for the self-administered questionnaire are reported in Appendix S2.

### Self-reported vaccination status

Among paramedical HCW, only 18% reported SIV, 9% PIV, 11% reported both vaccinations and 59% reported having received neither (Figure S2). For medical HCW, those rates were 10%, 22%, 48% and 18%, respectively (Figure S2). Overall, vaccination rates of paramedical and medical HCW were, respectively, 30% and 58% for SIV, and 21% and 71% for PIV (P<0.0001). The vaccination rates were also calculated by hospital (Figure S3). The percentages of respondents who reported SIV coverage during the three previous flu seasons are shown in Table 1 and their 2009 vaccination rates in Figure 2.

**Figure 4:**
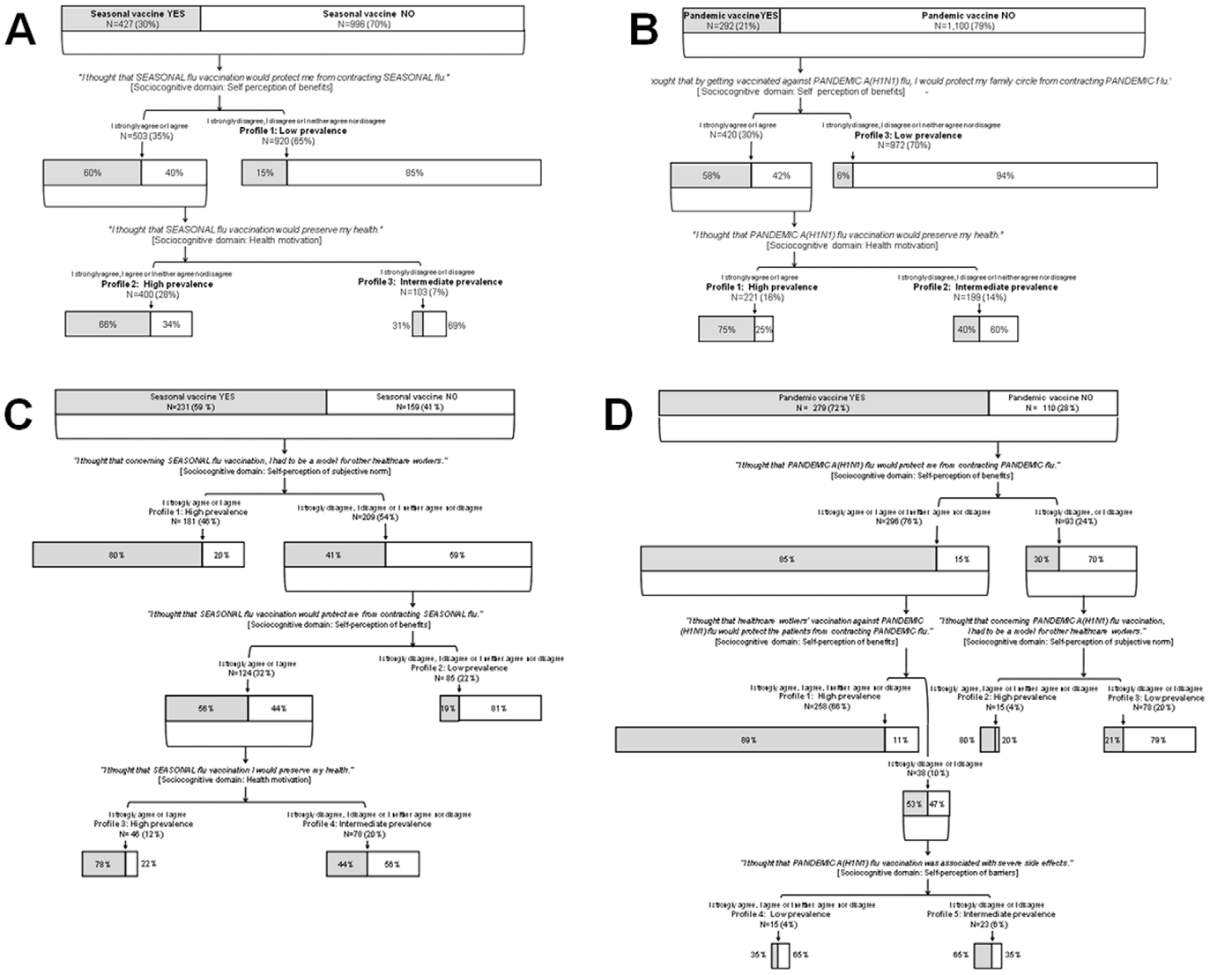
Classification-and-regression trees (CART) according to seasonal and pandemic A(H1N1) influenza vaccination status for paramedical and medical healthcare workers (HCW). The overall areas of the rectangles indicate the proportional sizes of the subgroup relative to the root population of HCW. Shaded areas represent the percentages of HCW in each subgroup that were actually vaccinated. The entire population was divided into subgroups based on the statements (reported in italics) that best discriminated between vaccinated and nonvaccinated HCW. At the termination and for each analysis, HCW were subdivided into profiles with high, intermediate and low prevalences of vaccination. Panel A: Seasonal influenza vaccination of paramedical HCW (N = 1,423). Panel B: Pandemic influenza vaccination of paramedical healthcare workers (N = 1,392). Panel C: Seasonal influenza vaccination of medical healthcare workers (N = 390). Panel D: Pandemic influenza vaccination of medical healthcare workers (N = 389).

### Factors associated with SIV and PIV

Multivariate analysis retained five factors as being associated with higher SIV rates (Table 2): older age, prior SIV, working in an ICU/ED, being a medical HCW and the hospital they worked in. Those five factors and working during day and alternating day/night shifts were significantly associated with higher PIV rates. There is certain amount of variation between hospitals as the effect sizes of the categories of that factor are statistically different from the reference one (“Beaujon”).

### HCWs' SIV and PIV profiles

Confirmatory factor analyses performed on S and A questionnaires showed that 8 of the 10 initial domains were identified. Items related to “self-perception of susceptibility” and “self-perception of severity” were confounded in a unique factor, as were “self-perception of behavioral norm” and “self-perception of subjective norm”.

Figures S4, S5, S6, and S7 report univariate analysis results of individual sociocognitive factors associated with status for both vaccinations and both HCW populations. Figure 3 shows the responses to the statement assessing perception of the vaccinations' benefit-risk balances.

The statements that best discriminated between vaccinated and nonvaccinated paramedical HCW were similar for SIV and PIV, and referred only to self-perception of benefits (i.e., to protect “oneself” (SIV) or “the family circle” (PIV)), and health motivation. Figure 4 Panels A and B show the final trees.

Considering SIV, willing to be a role model for their colleagues was the statement that best discriminated between vaccinated and nonvaccinated medical HCW. Among those 46% willing to be a role model for their colleagues, 80% had received SIV. For the 54% of medical HCW not considering themselves a role model, the statements best discriminating between vaccinated and nonvaccinated were the same as for paramedical HCW, i.e., “protect oneself” and “health motivation”. For PIV of medical HCW, discriminatory domains were self-perception of benefits, i.e., “oneself” or “patients”, self-perception of subjective norm, i.e., being a role model, and self-perception of barriers i.e., “severe side effects”. Figure 4 Panels C and D show the final trees.

Profiles of the population with at least one previous flu vaccination are reported for both vaccinations (Figures S8 and S9).

## Discussion

The results of this large multicenter study in France highlight key issues implicated in vaccination decision-making by HCW during the unprecedented 2009 pandemic alert. Vaccination rates differed according to vaccination type and occupational category: 30% and 58% (SIV) and 21% and 71% (PIV) for paramedical and medical HCW, respectively. A striking finding is that this decision-making involved limited and mainly self-centered sociocognitive dimensions, mainly “self-perception of benefits”, “health motivation” and, only for medical HCW, “self-perception of subjective norm”. The statements best discriminating between vaccinated and nonvaccinated paramedical HCW were similar for both vaccinations and concerned only self-perceived benefits, i.e., to protect “oneself” (SIV) or “the family circle” (PIV), and health motivation. Considering SIV, willing to be a role model was the statement that best discriminated between vaccinated and nonvaccinated medical HCW. Among those 46% willing to be a role model, 80% had received SIV. For the 54% of medical HCW not considering themselves a role model, the statements best discriminating between vaccinated and nonvaccinated were the same as for paramedical HCW, i.e., “protect oneself” and “health motivation”. Concerning PIV of medical HCW, discriminatory domains were perceived benefit effects for oneself or patients, being a role model or perceived barriers (side effects).

The A(H1N1) pandemic refocused attention on HCW as a priority group for vaccination to reduce their occupational risk of infection [6], its related absenteeism that might impact care delivery^5^ and to limit their role as vectors [22]. Hollmeyer et al. identified two major reasons for vaccination nonacceptance: a wide range of misconceptions about flu infection and related risks for patients, including the potential risk of transmission by HCW to their patients, and vaccination effectiveness; and a lack of convenient access to vaccination [13].

The US Healthcare Infection Control Practices Advisory Committee and the Advisory Committee on Immunization Practices recommend five components to improve HCWs' immunization rates: education and promotion, improved access, legislation and regulation, measurement and feedback, and role models [23]. However, the appropriate design and components of such multifaceted campaigns are unknown. In a survey involving 418 American centers, only free vaccination, adequate staff and resources, and education of targeted HCW groups predicted institutional vaccination rates [24]. Our healthcare group's 2009–2010 SIV–PIV campaign combined the first 4 (education and promotion, free and convenient vaccination, real-time feedback on the vaccination rates to each department, involvement of all hospitals' leaders and administration support) of those five recommended components, as French legislation and regulation cannot impose flu vaccination. Nevertheless, our vaccination rates remained as low as previously described in Europe [8], with only 11% of paramedical HCW and 48% of medical HCW having received both vaccinations.

A variety of factors have inconsistently been associated with HCWs' influenza-vaccination acceptance in previous studies, including individual and occupational characteristics, previous flu-immunization practices, and individual cognitive determinants [13]. Consistent with previous findings [25], 5 factors (older age, prior influenza vaccination, working in an ICU/ED, the hospital they worked in and being a medical HCW) predicted both vaccinations herein. However, our use of a sociocognitive approach and segmentation analysis provides new insights into interpreting decision-making and planning future vaccination campaigns.

Although the perceived benefit/risk-balance difference between vaccinated and nonvaccinated HCW supports the need for campaigns to address the misconceptions, those misunderstandings might be the reason for or the consequences of accepting or refusing flu vaccination. Indeed, reduction of cognitive dissonance, also called “rationalization” (i.e., people use of strategies to align their behavior with unconscious cognitions), is one of the most influential and extensively studied concepts in social psychology [26]. According to that theory, a paramedical HCW who refused vaccination assumes this refusal by espousing the most common misconceptions; importantly, this concept suggests that interventions, e.g. vaccination campaign, might reinforce misconceptions and paradoxically be counterproductive (reactance phenomenon) [27].

Our observations demonstrated that vaccination acceptance among paramedical HCW is mainly a self-centered concept. Therefore, approaches aiming at targeting personal benefits of immunization might be more successful than campaigns focused on preventing absenteeism and transmission to patients. Medical HCWs' decisions were marked by professionalism (being a role model and patient protection). These results plead strongly for campaigns targeting paramedical and medical HCW separately. Professional values develop largely through an informal process of socialization and training, and, thus, cannot be addressed in a vaccination campaign [28]. However, evidence from the medical and sociological literature suggests that the role model could play a pivotal part in changing human behavior [29]–[30].

Decision-making by previously vaccinated HCW was more complex but remained mainly self-centered, though it might be hypothesized that decision-making could rely on better understanding of or adherence to the recommendations. However, this analysis combined medical and paramedical populations, and our study was not designed to determine what led those HCW to be vaccinated for the first time.

Among the sociocognitive domains not involved in decision-making, “perceived barriers” and “external influence” deserve particular attention. Two themes were continuously raised by the French media during the 2009–2010 campaign, the safety of the pandemic vaccine and the noninvolvement of general practitioners in the information-and-immunization efforts [31]. Nevertheless, “external influences” was not identified as a key to decision-making in any of the models, and the fear of side effects was a consideration only for PIV by a few medical HCW.

This study has several strengths. First, its multicenter design with complementary hospitals in a same healthcare group and a high response rate strengthen its representativeness. Second, the large sample size allowed separate regression-tree analyses for medical and paramedical HCW. Third, 10 cognitive domains potentially involved in this complex decision were considered concurrently and not treated as isolated entities, as was done previously. Finally, the study was performed after the pandemic alert, which allowed dispassionate investigation of both vaccinations.

This study also has limitations. First, the prevalence of A(H1N1) influenza did not reach the anticipated pandemic state, with post-outbreak seropositivity rates ranging from 10 to 25% [32]–[35], and the prevalence of seasonal influenza remained lower than expected during the 2009–2010 winter. Even if our questionnaire was designed to assess HCWs' perceptions during the vaccination campaign, and not at the time of the study, we cannot exclude that the actual incidence of both diseases had impacted HCWs' responses to the items included in the CART analysis. Second, the worldwide extrapolation of our results is questionable. However, low vaccination rates and ineffective immunization campaigns have been reported in most parts of the world over three decades. Third, influenza vaccination was self-reported. Moreover, the lower response rate of medical HCW may reflect difficulty reaching them during the study days because of their activities, but we cannot exclude that some of them chose not to respond because of the topic's sensitivity. Then, given the nonlongitudinal study design, recall bias cannot be firmly ruled out. Finally, decision-making may be influenced by many factors not considered in the behavioral models.

Ours is the first study to offer a global comprehensive picture of decision-making for vaccination acceptance, a major challenge worldwide, by a large and diversified care group whose multifaceted campaign combined all the recommended components except specific legislation or regulation. We found that, among numerous well-recognized cognitive factors, only a few were involved in deciding to be vaccinated or not. Despite the important role played by professionalism in the medical community, vaccination acceptance is mainly a self-centered act. A multifaceted campaign without specific legislation or regulation policy might not be able to reach an efficient vaccination rate.

## Supporting Information

Figure S1 **Distributions of participants according to hospital and healthcare workers category.** HCW = healthcare workers. Occupations were unknown for 96 HCW from hospitals 1–5 (17, 47, 9, 8, and 15, respectively).
(TIF)

Figure S2 **Prevalences of seasonal and pandemic A(H1N1) influenza vaccinations of paramedical and medical healthcare workers.** SIV = seasonal vaccination. PIV = pandemic A(H1N1) vaccination. HCW = healthcare workers. Respective SIV and PIV rates were 30% and 58% for Paramedical HCW and 21% and 71% for Medical HCW (P<0.0001 for both vaccinations). Data were missing for 47 paramedical and 9 HCW.
(TIF)

Figure S3 **Prevalences of seasonal and pandemic A(H1N1) influenza vaccinations for paramedical and medical healthcare workers in each hospital.** HCW = healthcare workers. SIV = seasonal vaccination. PIV = pandemic A(H1N1) vaccination.
(TIF)

Figure S4 **Comparisons of individual sociocognitive factors between paramedical healthcare workers vaccinated (□) or nonvaccinated (▪) against seasonal influenza.**
(TIF)

Figure S5 **Comparisons of individual sociocognitive factors between paramedical healthcare workers vaccinated (□) or nonvaccinated (▪) against pandemic A(H1N1) influenza.**
(TIF)

Figure S6 **Comparisons of individual sociocognitive factors between medical healthcare workers vaccinated (□) or nonvaccinated (▪) against seasonal influenza.**
(TIF)

Figure S7 **Comparisons of individual sociocognitive factors between medical healthcare workers vaccinated (□) or nonvaccinated (▪) against pandemic A(H1N1) influenza.**
(TIF)

Figure S8 **Classification-and-regression tree according to seasonal influenza vaccination status for healthcare workers with at least one previous influenza vaccination (n = 864).** The overall areas of the rectangles indicate the proportional sizes of the subgroup relative to the root population of HCW. Shaded areas represent the percentages of HCW in each subgroup that were actually vaccinated. The entire population was divided into subgroups based on the statements (reported in italics) that best discriminated between vaccinated and nonvaccinated HCW. At the termination and for each analysis, HCW were subdivided into profiles with high, intermediate and low prevalences of vaccination.
(TIF)

Figure S9 **Classification-and-regression tree according to pandemic A(H1N1) influenza vaccination status for healthcare workers with at least one previous influenza vaccination (n = 854).** The overall areas of the rectangles indicate the proportional sizes of the subgroup relative to the root population of HCW. Shaded areas represent the percentages of HCW in each subgroup that were actually vaccinated. The entire population was divided into subgroups based on the statements (reported in italics) that best discriminated between vaccinated and nonvaccinated HCW. At the termination and for each analysis, HCW were subdivided into profiles with high, intermediate and low prevalences of vaccination.
(TIF)

Methods S1 **Methods long version: passages in the main manuscript were dimmed to facilitate reading of new information.**
(DOC)

Table S1 **Characteristics of the five hospitals participating in the INFLUENCE-A study.** Data are number (%). ICU = intensive care unit. ED = emergency Department. *Hospital locations in the metropolitan Paris area: Colombes (Louis-Mourier Hospital), Villiers-le-Bel (Charles-Richet Hospital), Clichy (Beaujon Hospital), and Paris inner city (Bichat and Bretonneau Hospitals). †Including medical, surgical, medical–surgical, cardiac and neurologic units. ‡Including 77 pediatric beds (newborns and children). §Total number of full-time positions on 1 October 2009. ¶Number of cases confirmed by specific polymerase chain reaction on respiratory samples between 1 July 2009 and 30 April 2010.
(DOC)

Table S2 **Definitions of sociocognitive domains and individual sociocognitive factors with corresponding self-administered questionnaire statements.** PMHCW = paramedical healthcare workers (nurses, nurses' aides, physiotherapists and orderlies). MHCW = medical healthcare workers (doctors, medical students and midwives) based on the authorization to prescribe. *Section 4 (seasonal-influenza vaccination) and Section 5 (pandemic A(H1N1) influenza) of the self-administered questionnaire (see Appendix S1). The statement no. 34 was “I thought that the benefit of flu vaccination was greater than its related risks” and thus relates to 2 domains, self-perception of benefits and barriers.
(DOC)

Table S3 **Response rates in each participating center.** Response rates are expressed as numbers of responses/total numbers of eligible healthcare workers (%). HCW = healthcare workers.
(DOC)

Appendix S1 **English version of the questionnaire used in the INFLUENCE A study.**
(DOC)

Appendix S2 **Numbers of missing data per item for the self-administered questionnaire.**
(DOC)
