# Supplementary figures and images for: Personal Decision-Making Criteria Related to Seasonal and Pandemic A(H1N1) Influenza-Vaccination Acceptance among French Healthcare Workers
Source: PLoS One. 2012 Jul 27;7(7):e38646. doi: 10.1371/journal.pone.0038646 (PMC3407215; doi:10.1371/journal.pone.0038646)

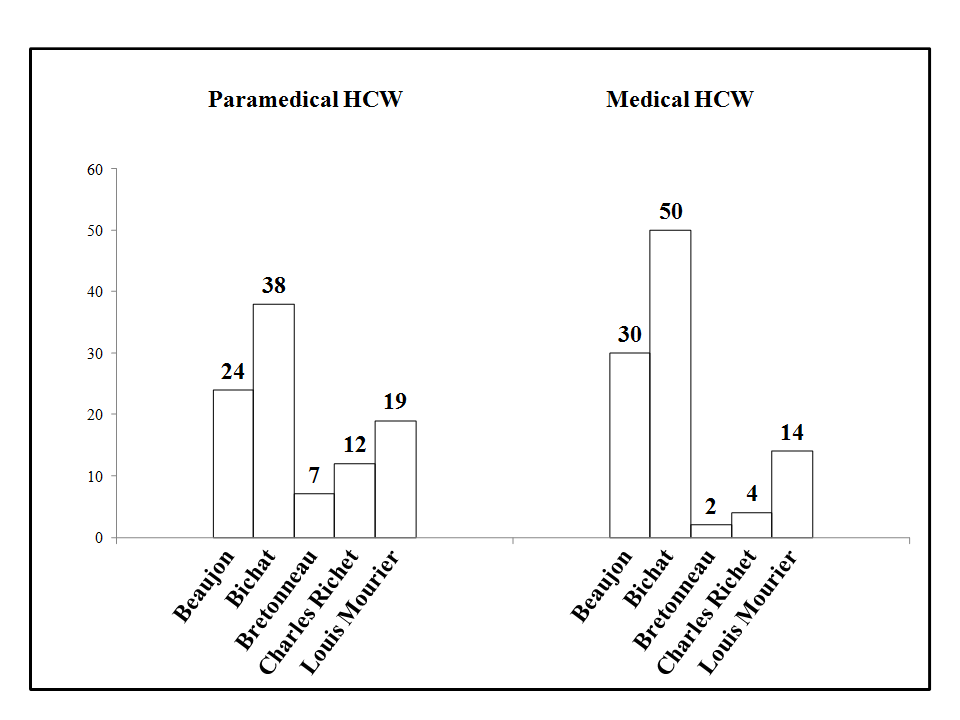

Supplement: Figure S1 — Distributions of participants according to hospital and healthcare workers category. HCW = healthcare workers. Occupations were unknown for 96 HCW from hospitals 1–5 (17, 47, 9, 8, and 15, respectively). (TIF) [file pone.0038646.s001.tif]

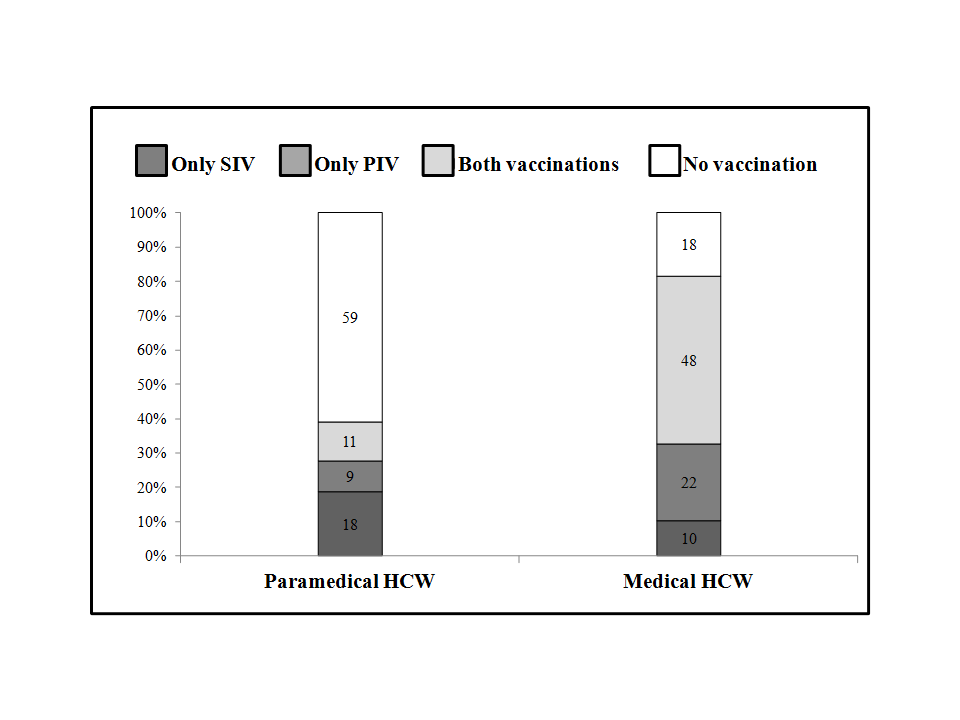

Supplement: Figure S2 — Prevalences of seasonal and pandemic A(H1N1) influenza vaccinations of paramedical and medical healthcare workers. SIV = seasonal vaccination. PIV = pandemic A(H1N1) vaccination. HCW = healthcare workers. Respective SIV and PIV rates were 30% and 58% for Paramedical HCW and 21% and 71% for Medical HCW (P<0.0001 for both vaccinations). Data were missing for 47 paramedical and 9 HCW. (TIF) [file pone.0038646.s002.tif]

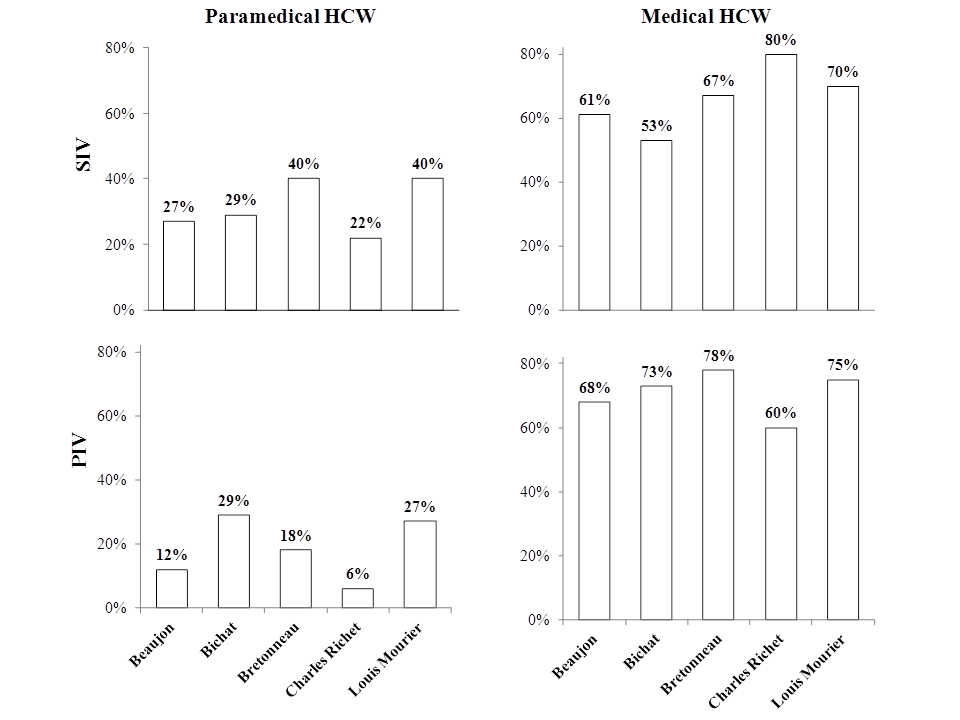

Supplement: Figure S3 — Prevalences of seasonal and pandemic A(H1N1) influenza vaccinations for paramedical and medical healthcare workers in each hospital. HCW = healthcare workers. SIV = seasonal vaccination. PIV = pandemic A(H1N1) vaccination. (TIF) [file pone.0038646.s003.tif]

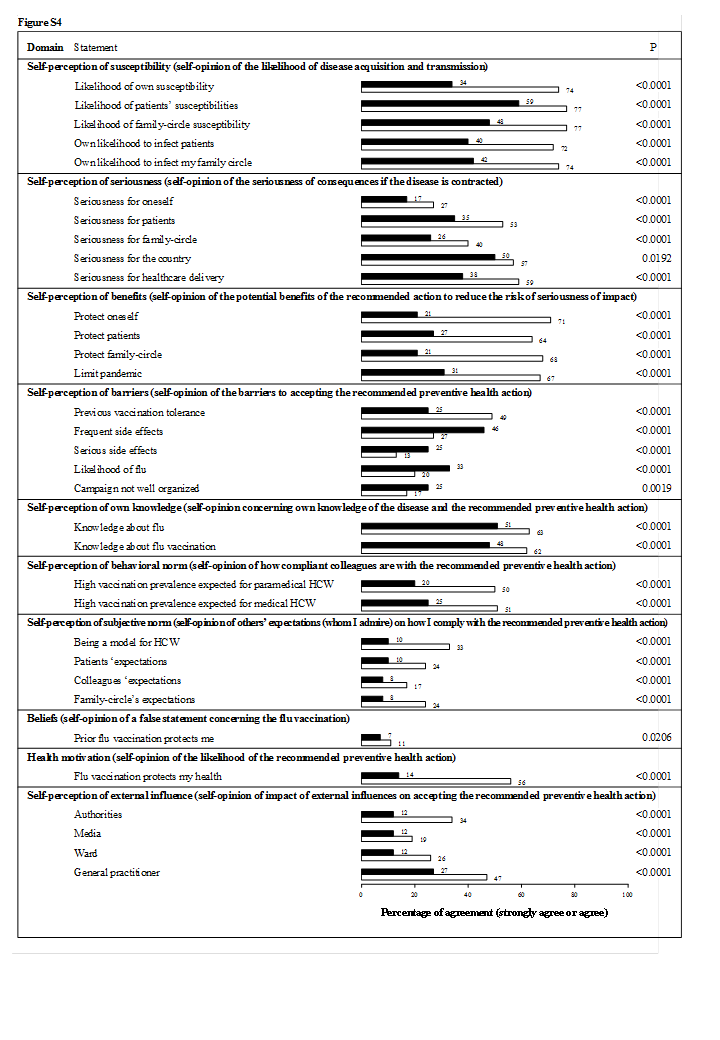

Supplement: Figure S4 — Comparisons of individual sociocognitive factors between paramedical healthcare workers vaccinated (□) or nonvaccinated (▪) against seasonal influenza. (TIF) [file pone.0038646.s004.tif]

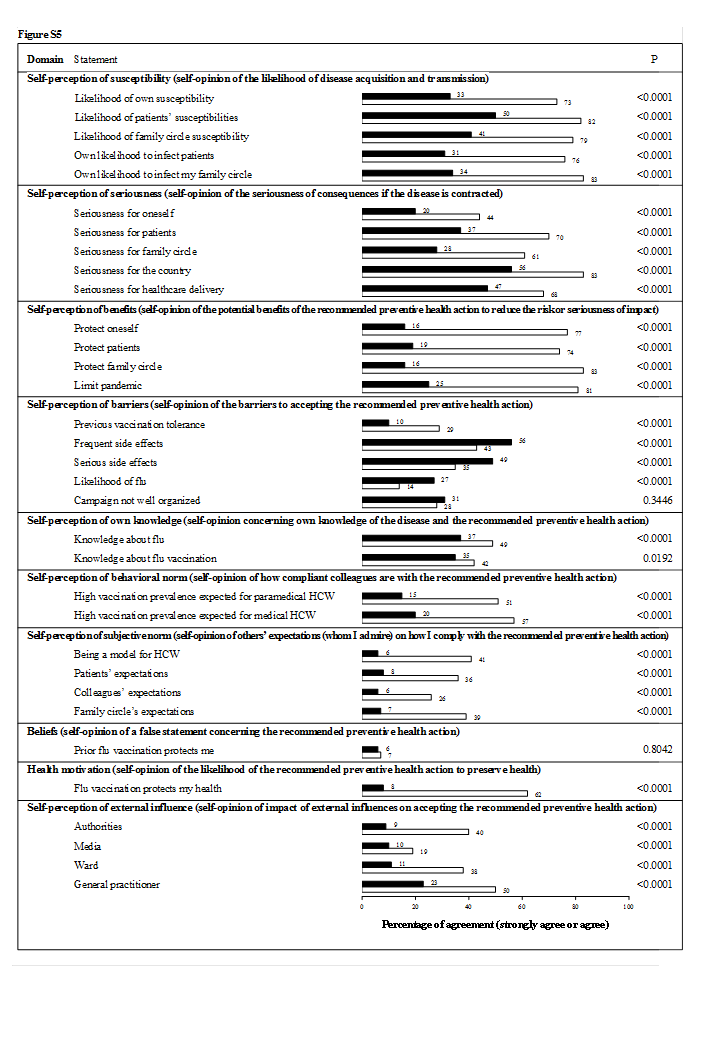

Supplement: Figure S5 — Comparisons of individual sociocognitive factors between paramedical healthcare workers vaccinated (□) or nonvaccinated (▪) against pandemic A(H1N1) influenza. (TIF) [file pone.0038646.s005.tif]

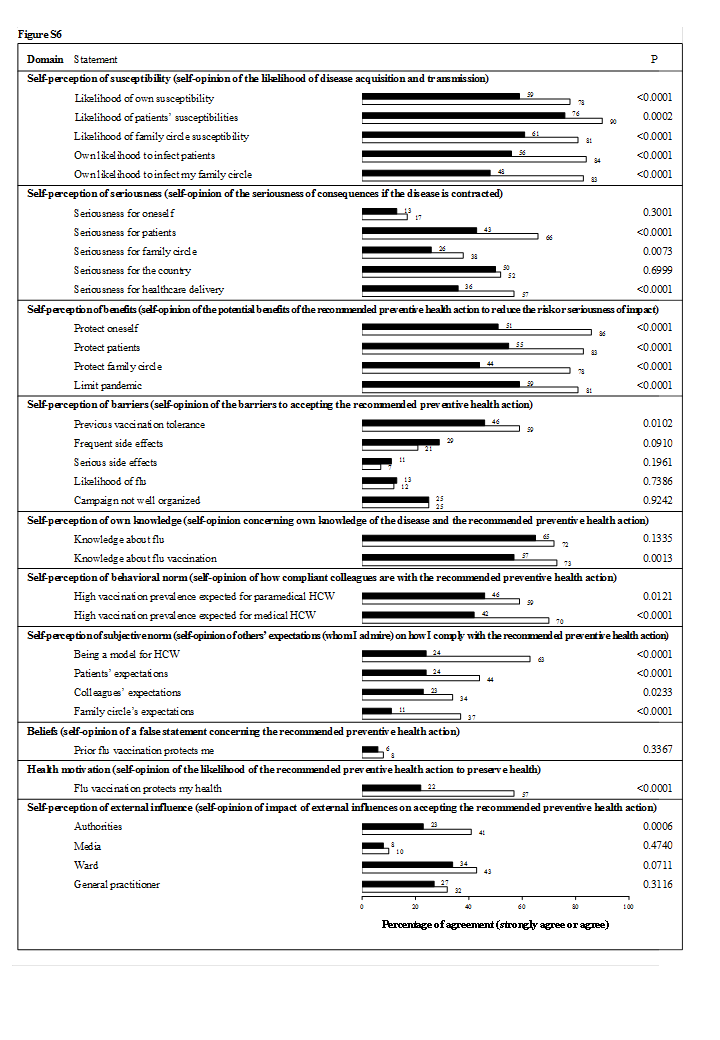

Supplement: Figure S6 — Comparisons of individual sociocognitive factors between medical healthcare workers vaccinated (□) or nonvaccinated (▪) against seasonal influenza. (TIF) [file pone.0038646.s006.tif]

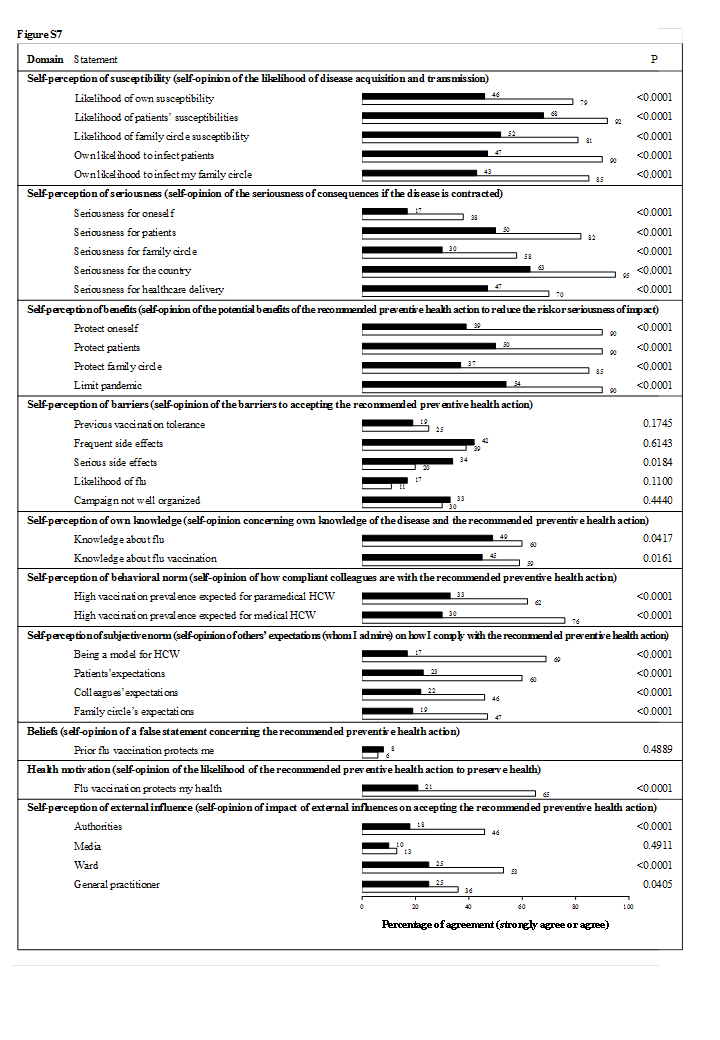

Supplement: Figure S7 — Comparisons of individual sociocognitive factors between medical healthcare workers vaccinated (□) or nonvaccinated (▪) against pandemic A(H1N1) influenza. (TIF) [file pone.0038646.s007.tif]

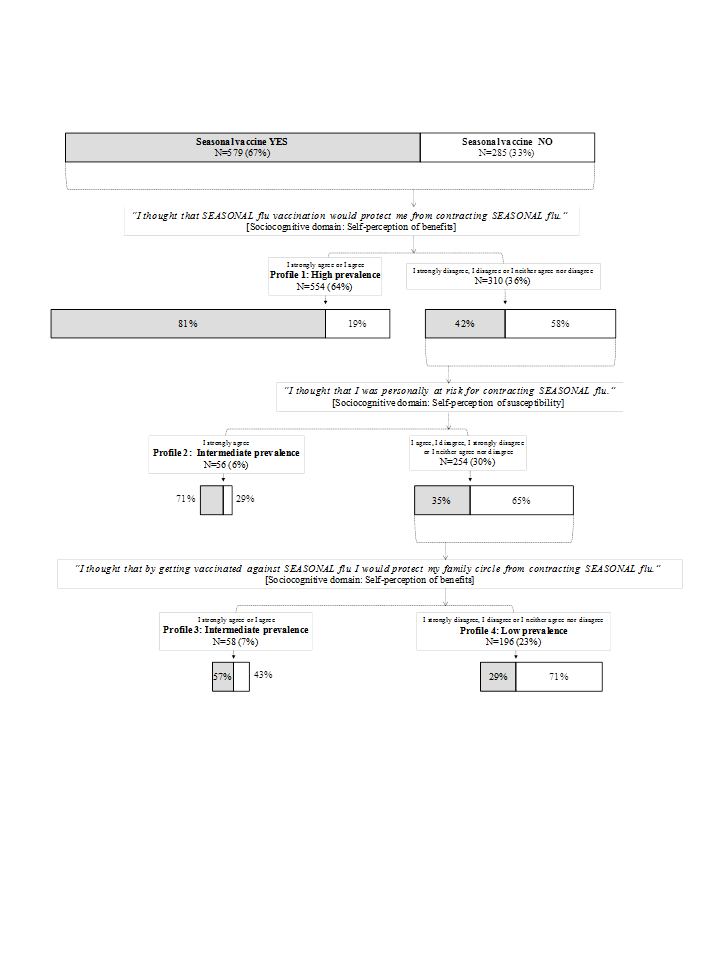

Supplement: Figure S8 — Classification-and-regression tree according to seasonal influenza vaccination status for healthcare workers with at least one previous influenza vaccination (n = 864). The overall areas of the rectangles indicate the proportional sizes of the subgroup relative to the root population of HCW. Shaded areas represent the percentages of HCW in each subgroup that were actually vaccinated. The entire population was divided into subgroups based on the statements (reported in italics) that best discriminated between vaccinated and nonvaccinated HCW. At the termination and for each analysis, HCW were subdivided into profiles with high, intermediate and low prevalences of vaccination. (TIF) [file pone.0038646.s008.tif]

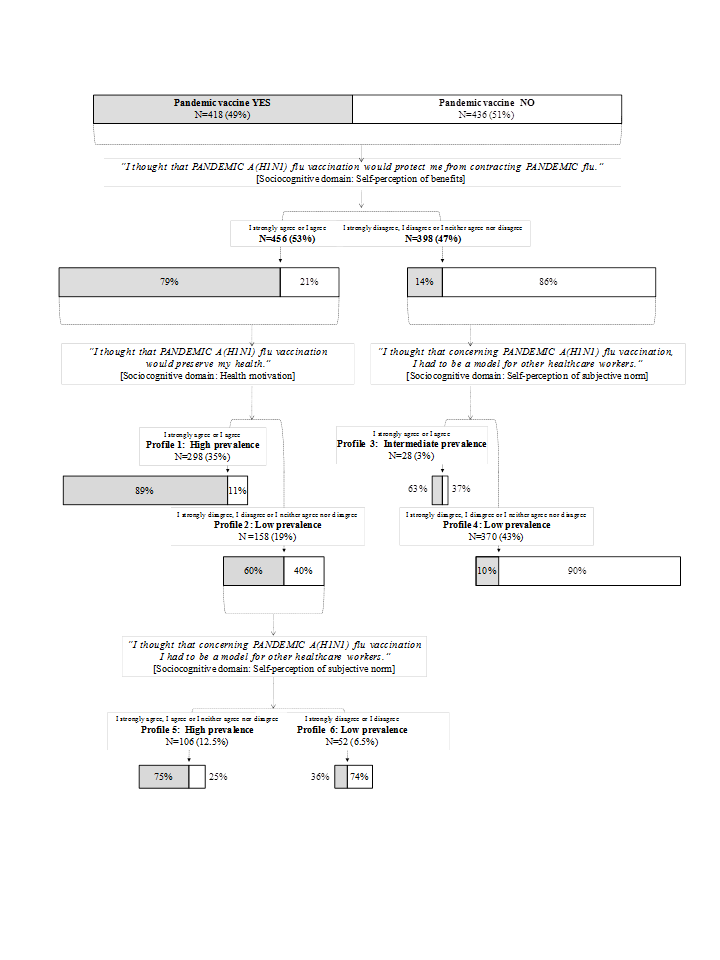

Supplement: Figure S9 — Classification-and-regression tree according to pandemic A(H1N1) influenza vaccination status for healthcare workers with at least one previous influenza vaccination (n = 854). The overall areas of the rectangles indicate the proportional sizes of the subgroup relative to the root population of HCW. Shaded areas represent the percentages of HCW in each subgroup that were actually vaccinated. The entire population was divided into subgroups based on the statements (reported in italics) that best discriminated between vaccinated and nonvaccinated HCW. At the termination and for each analysis, HCW were subdivided into profiles with high, intermediate and low prevalences of vaccination. (TIF) [file pone.0038646.s009.tif]
