# Supplementary material for: Personal Decision-Making Criteria Related to Seasonal and Pandemic A(H1N1) Influenza-Vaccination Acceptance among French Healthcare Workers
Source: PLoS One. 2012 Jul 27;7(7):e38646. doi: 10.1371/journal.pone.0038646 (PMC3407215; doi:10.1371/journal.pone.0038646)
Supplement: Methods S1 — Methods long version: passages in the main manuscript were dimmed to facilitate reading of new information. (DOC) [file pone.0038646.s010.doc]

**Supporting information text**

**methods s1**

**Personal decision-making criteria related to seasonal and pandemic A(H1N1) influenza-vaccination acceptance among French healthcare workers**

Lila Bouadma*, M.D., Ph.D., François Barbier, M.D., Lucie Biard, M.D., Marina Esposito-Farèse, Ph.D., Bertrand Le Corre, R.N., Annick Macrez, R.N., Laurence Salomon, M.D., Ph.D., Christine Bonnal, M.D., Caroline Zanker, M.D., Christophe Najem, Pharm.D., Bruno Mourvillier, M.D., Jean Christophe Lucet, M.D., Ph.D., Bernard Régnier, M.D., Michel Wolff, M.D., Ph.D., Florence Tubach, M.D., Ph.D., for the INFLUENCE-A Study Group

From the departments of Réanimation Médicale et des Maladies Infectieuses (Li.B., F.B., B.M., B.R., M.W.), Département d'Epidémiologie Biostatistique et Recherche Clinique (Lu.B., F.T.), Direction de la Qualité et Gestion des Risques (B.L.C., A.M.), Unité d’Hygiène et de Lutte contre l’Infection Nosocomiale (J.C.L), all in Hôpital Bichat–Claude-Bernard, Paris; Département de Santé Publique (L.S.), Hôpital Louis-Mourier, Colombes; Unité d’Hygiène et de Lutte contre l’Infection Nosocomiale (C.B.), Hôpital Bretonneau, Paris; Urgences Médicales Urgences Chirurgicales (C.Z.), Hôpital Beaujon, Clichy; Service de Pharmacie/Hygiène (C.N.), Hôpital Charles-Richet, Villiers-le-Bel; all from the Hôpitaux Universitaires Paris Nord–Val de Seine, Assistance Publique–Hôpitaux de Paris, France ; Université Paris Diderot - Sorbonne, Paris, France (J.C.L., B.R., M.W., F.T.) ; EA 3964 (F.B. and M.W.), Université Paris Diderot, Paris, France ; CIE 801, Institut National de la Santé et de la Recherché Médicale, Paris, France (F.T.).

**Address all correspondence to** Dr. Lila Bouadma, Réanimation Médicale et des Maladies Infectieuses, Hôpital Bichat-Claude Bernard, 46, rue Henri-Huchard, 75877 Paris Cedex 18, France. Tel: +33 (0) 1 40 25 77 03; Fax: +33 (0) 1 40 25 88 37; E-mail:[lila.bouadma@bch.aphp.fr](mailto:lila.bouadma@bch.aphp.fr).

**Study characteristics**

On a given day, 8,367 HCW in a five-hospital French university healthcare group located in Northern Paris and its suburbs participated in a cross-sectional study. This group has 2,622 beds with diversified and complementary activities (see Supplementary Material **Table S1)**. Bichat–Claude-Bernard Hospital, a tertiary-care center for infectious diseases and pandemic influenza, has 987 beds; the 472-bed Beaujon Hospital has mostly surgical activities; Charles-Richet (472 beds) and Bretonneau (205 beds) Hospitals are dedicated to geriatric care; Louis-Mourier Hospital has 486 beds, including acute care, rehabilitation and long-term–care units.

HCW are defined as medical (doctors, medical students and midwives) and paramedical (nurses, nurses’ aides, physiotherapists and orderlies) based on the authorization to prescribe.

In 2009, this group conducted a multifaceted PIV and SIV campaign, combining education and encouragement, free and convenient vaccination, real-time feedback on vaccination rates to each department, involvement of all hospital leaders and administration support (see Supplementary Material).

Our healthcare group established a committee, involving administrative staff, the infection control and occupational medicine units, the infectious disease department, and the emergency and medical intensive care units, to manage the influx of patients and the vaccination campaign. Committee meetings were held at least once a week throughout the campaign. Various approaches (conferences with interactive discussions, posters placed throughout the hospitals, information and announcements via email and intranet websites, and emailing head nurses and head physicians of all departments) were used to inform HCW about the two influenza viruses (seasonal and A(H1N1)) and their respective vaccines. The Committee was informed weekly of the vaccination rate and provided feedback in real time to all departments. Free SIV and PIV were offered to all HCW and were administered free-of-charge from 1 September to 20 October 2009, directly in the ward or in the occupational medicine unit. Vaccines were administered by physicians and nurses from infectious disease, infection control and occupational medicine departments during large windows covering day and night shifts. Both vaccinations were subsequently available during the whole period of pandemic influenza.

The survey was conducted over two consecutive weeks, between 25 June and 8 July 2010, over 24 hours the same weekday in each hospital. All HCW working in inpatient wards the study day were eligible to participate, regardless of their shift (night or day). HCW not directly in charge of patients (secretaries and technical, laboratory and administrative staff) and those from outpatient clinics, operating rooms and radiology departments were not included. A study-dedicated monitor individually distributed anonymous self-administered questionnaires to all HCW present in each hospital over the defined 24-hour period, emphasizing the questionnaire’s anonymous nature and encouraging HCW to complete it before placing it in an anonymous envelope that the monitors collected the same day.

Participants were informed by posters announcing the study in all concerned wards 1 week before the study day. All ward heads were informed via emails before the study day.

The Institutional Review Board (Comité d’Ethique du Groupe Hospitalo-Universitaire Paris-Nord, IRB no. 00006477) approved the study protocol and waived the need for written informed consent of the participants.

**The questionnaire**

Four investigators devised the questionnaire (Supplementary Appendix 1).

Part of the questionnaire addresses demographic, personal, family and professional characteristics, history of influenza vaccination, and current SIV and PIV status.

The rest of the questionnaire was developed using an iterative process and was based on sociocognitive models and theories adapted to the context of influenza vaccination, and took into account questionnaires from previous research in this field [13]. The sociocognitive models and theories were developed to explain, predict or influence behavior. None of these models or theories, by itself, can entirely explain a behavior and they sometimes overlap. Current models and theories that help explain human health-related behavior targeted different levels: individual (intrapersonal), interactions between individuals (interpersonal), the institution and the community [14].

Among the community-level models, the Theory of Ecological Perspective is based on two key ideas, i.e., 1) behavior is viewed as being affected by multiple levels of influence, and 2) behavior influences and is influenced by the social environment [15].

Intrapersonal factors are individual characteristics that affect behavior, such as knowledge, attitudes, beliefs and personality traits. They are contained in sociocognitive models, such as the Health Belief Model (HBM) [16] and the Theory of Planned Behavior [17] used frequently to investigate health behavior and influenza-vaccination acceptance.

Interpersonal factors include interpersonal processes between two or more people; they range from fleeting to enduring. They may be based on family, love, friendship, work and position, or some other types of social commitment. Individuals can be influenced by others (i.e., family, friends and peers) in their social environments and, notably, health behavior is often influenced by peer-group pressure. The Social Learning Theory states that people learn not only through their own experience, but also by observing others’ actions and the results of those actions, as well as through role modeling [18].

This part of the questionnaire finally consists of 33 statements, reflecting sociocognitive theories applied to health behaviors, adapted to the context of influenza vaccination and addressing potential individual sociocognitive factors of influenza-vaccination acceptance (see Supplementary Material),^20-25^ grouped into 10 cognitive dimensions: 1) self-perception of susceptibility (self-opinion of the likelihood of disease acquisition and transmission, n=5); 2) self-perception of seriousness (self-opinion of the seriousness of consequences if the disease is contracted, n=5); 3) self-perception of benefits (self-opinion of the potential benefits of the recommended preventive health action to reduce the risk or seriousness of impact, n=4); 4) self-perception of barriers (self-opinion of the barriers to accepting the recommended preventive health action, n=5); 5) self-perception of own knowledge (self-opinion concerning own knowledge of the disease and the recommended preventive health action, n=2); 6) self-perception of behavioral norm (self-opinion of how compliant colleagues are with the recommended preventive health action, n=2); 7) self-perception of subjective norm (self-opinion of the expectations of others (whom I admire) on how I comply with the recommended preventive health action, n=4); 8) beliefs (self-opinion of a false statement concerning the recommended preventive health action, n=1); 9) health motivation (self-opinion of the likelihood of the recommended preventive health action to preserve health, n=1); and 10) self-perception of external influences (self-opinion of impact of external influences on accepting the recommended preventive health action, n=4).

According to the dimension, when pertinent, a statement referred to the HCW, his/her family circle or patients (see supplementary **Table S2** for definitions and **Appendix 1** for related questionnaire items). Another more global item assessing the perception of the risk-benefit balance was added: “I thought that the benefit of flu vaccination was greater than its related risks”. The 34 items were used twice in the final questionnaire, first to address SIV and then PIV. HCW had to rate their degree of agreement with each statement with a Likert 5-point scale (from strongly agree to strongly disagree).

The two parts of the questionnaire were tested with a representative medical and non-medical HCW panel, and suggestions raised on wording and format were incorporated into the final version. That version of the self-administered questionnaire was approved by four questionnaire creators (see **Appendix 1** for the English translation of the questionnaire).

**Establishment of the database**

Questionnaire responses were entered using a double-keyboarding procedure. Given the anonymity of the survey, missing data were not obtainable a posteriori. All uncertain responses were reviewed by the investigators (Li.B., F.B., and Lu.B.) and resolved whenever possible. Non-resolvable queries and equivocal responses were considered missing data for statistical analysis (numbers of missing data per item for the self-administered questionnaire are reported in **Appendix 2**).

The following hospital characteristics were obtained from administrators: numbers of beds per ward category, total numbers of medical and paramedical HCW employed during the 2009–2010 winter, numbers of hospitalizations and Emergency Department visits in 2009. Data on admissions, ICU stays and deaths of inpatients with confirmed A(H1N1)influenza were provided by each hospital’s Infection Control Units, and the national public health agency (Institut de Veille Sanitaire, Saint-Maurice, France). A(H1N1) cases not confirmed by molecular assays on respiratory samples (i.e. suspected cases) were not retained **(Table S1)**.

**Statistical analyses**

The sample size was the number of HCW in the 5 hospitals who completed the questionnaire. The denominator of the participation rate (i.e., medical and paramedical HCW present on the study day) was obtained from each ward’s administrative staff. Because response rates differed widely between medical and paramedical HCW, these categories were considered separately, thereby excluding from all the analyses the 96 HCW of unknown paramedical or medical status.

For each of the vaccination status SIV or PIV, factors associated were identified for the entire population by univariate analyses. Potential factors considered for both vaccination status were sex, age, prior SIV at least once during the three previous winters, work shift (day only, night only, or alternating day/night), living alone or with others, pregnancy, chronic disease (i.e., cardiac disease, cancer, history of stroke, chronic respiratory failure, diabetes mellitus, and/or immunosuppression), professional status (paramedical or medical HCW), and ward (intensive care unit (ICU) or emergency department (ED), acute medical (including obstetric and pediatric) or surgical, rehabilitation or long-term care, others or unknown). For multivaried analysis, logistic regressions were fitted for each vaccination status separately. Adjusting factors in the models were those significantly associated (P≤0.1) in univariate analyses or potentially important. For SIV the adjusting factors were pandemic vaccination, age, working hours, professional category, ward and center. For PIV the adjusting factors were seasonal vaccination, gender, age, working hours, professional category, ward, living alone, pregnancy and center. Final models were selected with stepwise procedures (backward, forward and both). All of these procedures gave the same results, comforting the selection of the models.

To identify homogeneous profiles of paramedical and medical HCW in terms of the individual sociocognitive items, these factors were subjected to a decision-tree–classification method based on recursive partitioning analysis. The endpoint-of-interest was whether or not the HCW chose vaccination. The classification-and-regression trees (CART)were performed with the “rpart” package,^19^ which implements CART^20^ ideas using R software v.2.12.^21^

Two distinct partitionings were done: one focusing on SIV (S Questionnaire items) and the other on PIV status (A Questionnaire items). Separate analyses were performed for paramedical and medical HCW. Analyses were repeated separately for all HCW with prior SIV combined.

For each partitioning process, the overall probability of the outcome (vaccinated or not) was estimated. Then, the entire population was divided into subgroups based on the items (out of the 33 sociocognitive determinants) that best reflected the probability of the outcome based on responses. This process continued with each subgroup until the added level of complexity could no longer be justified by efforts to validate. The additional item 34 on the perceived benefit risk of the vaccination was not included, given that it summarizes different cognitive dimensions.

Associations between perceived vaccination benefit/risk and current vaccination status were tested separately with the χ^2^ test for paramedical and medical HCW.

Individual sociocognitive factors were compared between vaccinated or nonvaccinated against type of influenza with the χ^2^-square test for medical and paramedical HCW.
