# Supplementary material for: Personal Decision-Making Criteria Related to Seasonal and Pandemic A(H1N1) Influenza-Vaccination Acceptance among French Healthcare Workers
Source: PLoS One. 2012 Jul 27;7(7):e38646. doi: 10.1371/journal.pone.0038646 (PMC3407215; doi:10.1371/journal.pone.0038646)
Supplement: Table S1 — Characteristics of the five hospitals participating in the INFLUENCE-A study. Data are number (%). ICU = intensive care unit. ED = emergency Department. *Hospital locations in the metropolitan Paris area: Colombes (Louis-Mourier Hospital), Villiers-le-Bel (Charles-Richet Hospital), Clichy (Beaujon Hospital), and Paris inner city (Bichat and Bretonneau Hospitals). †Including medical, surgical, medical–surgical, cardiac and neurologic units. ‡Including 77 pediatric beds (newborns and children). §Total number of full-time positions on 1 October 2009. ¶Number of cases confirmed by specific polymerase chain reaction on respiratory samples between 1 July 2009 and 30 April 2010. (DOC) [file pone.0038646.s011.doc]

**Table S1. Characteristics of the five hospitals participating in the INFLUENCE-A study**

| **Characteristic** | **Beaujon*** | **Bichat*** | **Bretonneau*** | **Charles-Richet*** | **Louis-Mourier*** | **Total** |
| --- | --- | --- | --- | --- | --- | --- |
| Study day | 8 July 2010 | 25 June 2010 | 1 July 2010 | 1 July 2010 | 28 June 2010 | – |
| Patient recruitment | Adult | Adult/elderly | Elderly | Elderly | Pediatric/adult | – |
| Beds | 472 | 987 | 205 | 472 | 486 | 2,622 |
| *ICU†* | 33 (7) | 70 (7.1) | – | – | 14 (2.9) | 117 (4.5) |
| *ED* | 10 (2.1) | 20 (2.0) | – | – | 8 (1.6) | 38 (1.4) |
| *Medical wards* | 165 (35) | 496 (50.3) | – | – | 262 (53.9) ‡ | 923 (35.2) |
| *Surgical wards* | 226 (47.9) | 297 (30.1) | – | – | 36 (7.4) | 559 (21.3) |
| *Obstetrics wards* | 38 (8.1) | 39 (4) | – | – | 56 (11.5) | 133 (5.1) |
| *Rehabilitation and long-term care* | – | 65 (6.6) | 205 (100) | 472 (100) | 110 (22.6) | 852 (32.5) |
| Healthcare workers§ | 2,551 | 3,679 | 235 | 339 | 1,563 | 8,367 |
| *Medical* | 509 (20) | 1,100 (29.9) | 8 (3.4) | 34 (10.0) | 512 (32.8) | 2,163 (25.9) |
| *Paramedical* | 2,042 (80.0) | 2,579 (70.1) | 227 (96.6 ) | 305 (90) | 1,051 ( 67.2) | 6,204 (74.1) |
| Inpatients (admissions) in 2009 | 30,806 | 63,511 | 6,678 | 1,755 | 33,465 | 136,215 |
| Outpatients admitted to the ED/day of 2009 | 30,125 | 67,160 | – | – | 59,860 | 158,045 |
| Inpatients with pandemic A(H1N1) influenza¶ | 5 | 100 | 0 | 0 | 6 | 111 |
| *ICU admissions* | 2 | 20 | – | – | 6 | 28 |
| *Deaths* | 0 | 5 | – | – | 1 | 6 |

**Table S1, footnote.**

Data are number (%). ICU=intensive care unit. ED=emergency Department. *Hospital locations in the metropolitan Paris area: Colombes (Louis-Mourier Hospital), Villiers-le-Bel (Charles-Richet Hospital), Clichy (Beaujon Hospital), and Paris inner city (Bichat and Bretonneau Hospitals). †Including medical, surgical, medical–surgical, cardiac and neurologic units. ‡Including 77 pediatric beds (newborns and children). §Total number of full-time positions on 1 October 2009. ¶Number of cases confirmed by specific polymerase chain reaction on respiratory samples between July 1^st^, 2009 and April 30^th^, 2010.
