# Supplementary material for: Personal Decision-Making Criteria Related to Seasonal and Pandemic A(H1N1) Influenza-Vaccination Acceptance among French Healthcare Workers
Source: PLoS One. 2012 Jul 27;7(7):e38646. doi: 10.1371/journal.pone.0038646 (PMC3407215; doi:10.1371/journal.pone.0038646)
Supplement: Table S2 — Definitions of sociocognitive domains and individual sociocognitive factors with corresponding self-administered questionnaire statements. PMHCW = paramedical healthcare workers (nurses, nurses' aides, physiotherapists and orderlies). MHCW = medical healthcare workers (doctors, medical students and midwives) based on the authorization to prescribe. *Section 4 (seasonal-influenza vaccination) and Section 5 (pandemic A(H1N1) influenza) of the self-administered questionnaire (see Appendix S1). The statement no. 34 was “I thought that the benefit of flu vaccination was greater than its related risks” and thus relates to 2 domains, self-perception of benefits and barriers. (DOC) [file pone.0038646.s012.doc]

**Table S2. Definitions of sociocognitive domains and individual sociocognitive factors with corresponding self-administered** questionnaire statements

| **Sociocognitive domain** | **Factor** | **Statement†** |
| --- | --- | --- |
| Self-perception of susceptibility - *Self-opinion of the likelihood of disease acquisition and transmission* | Likelihood of own susceptibility | 1 |
|  | Likelihood of patients’ susceptibilities | 2 |
|  | Likelihood of family circle susceptibility | 3 |
|  | Own likelihood to infect patients | 4 |
|  | Own likelihood to infect my family circle | 5 |
| Self-perception of seriousness - *Self-opinion of the seriousness of consequences if the disease is* | Seriousness for oneself | 6 |
| *contracted* | Seriousness for patients | 7 |
|  | Seriousness for family circle | 8 |
|  | Seriousness for the country | 9 |
|  | Seriousness for healthcare delivery | 10 |
| Self-perception of benefits*****- *Self-opinion of the potential benefits of the recommended preventive health* | Protect oneself | 11 |
| *action to reduce the risk or seriousness of impact* | Protect patients | 12 |
|  | Protect family circle | 13 |
|  | Limit pandemic | 14 |
| Self-perception of barriers*****- *Self-opinion of the barriers to accepting the recommended preventive health* | Previous vaccination tolerance | 15 |
| *action* | Frequent side effects | 16 |
|  | Serious side effects | 17 |
|  | Likelihood of flu | 18 |
|  | Campaign not well organized | 19 |
| Self-perception of own knowledge - *Self-opinion concerning own knowledge of the disease and the* | Knowledge about flu | 20 |
| *recommended preventive health action* | Knowledge about flu vaccination | 21 |
| Self-perception of behavioral norm - *Self-opinion of how compliant colleagues are with the recommended* | High vaccination rate expected for PMHCW | 22 |
| *preventive health action* | High vaccination rate expected for MHCW | 23 |
| Self-perception of subjective norm - *Self-opinion of the expectations of others (whom I admire) on how I* | Being a model for HCW | 24 |
| *comply with the recommended preventive health action* | Patients’ expectations | 25 |
|  | Colleagues’ expectations | 26 |
|  | Family circle’s expectations | 27 |
| Beliefs - *Self-opinion of a false statement concerning the recommended preventive health action* | Prior flu vaccination protects me | 28 |
| Health motivation - *Self-opinion of the likelihood of the recommended preventive health action to preserve health* | Flu vaccination protects my health | 29 |
| Self-perception of external influences - *Self-opinion of impact of external influences on accepting the* | Authorities | 30 |
| *recommended preventive health action* | Media | 31 |
|  | Ward | 32 |
|  | General practitioner | 33 |

**Table S2, footnote.**

PMHCW=paramedical healthcare workers (nurses, nurses’ aides, physiotherapists and orderlies). MHCW=medical healthcare workers (doctors, medical students and midwives) based on the authorization to prescribe. *****Section 4 (seasonal-influenza vaccination) and Section 5 (pandemic A(H1N1) influenza) of the self-administered questionnaire (see **Appendix 1:** p 28-31). The statement no. 34 was “I thought that the benefit of flu vaccination was greater than its related risks” and thus relates to 2 domains, self-perception of benefits and barriers.
