# Supplementary material for: Personal Decision-Making Criteria Related to Seasonal and Pandemic A(H1N1) Influenza-Vaccination Acceptance among French Healthcare Workers
Source: PLoS One. 2012 Jul 27;7(7):e38646. doi: 10.1371/journal.pone.0038646 (PMC3407215; doi:10.1371/journal.pone.0038646)
Supplement: Table S3 — Response rates in each participating center. Response rates are expressed as numbers of responses/total numbers of eligible healthcare workers (%). HCW = healthcare workers. (DOC) [file pone.0038646.s013.doc]

**Table S3. Response rates in each participating center**

| **Participants** | **1 Beaujon** | **2 Bichat** | **3 Bretonneau** | **4 Charles-Richet** | **5 Louis-Mourier** | **Total** |
| --- | --- | --- | --- | --- | --- | --- |
| All HCW | 489/614 (79.6) | 807/994 (81.2) | 130/173 (75.1) | 198/269 (73.6) | 353/386 (91.5) | 1,977/2,436 (81.2) |
| Paramedical HCW | 353/450 (78.4) | 558/709 (78.7) | 112/151 (74.2) | 175/254 (68.9) | 282/296 (95.3) | 1,480/1,860 (79.6) |
| Medical HCW | 119/164 (72.6) | 202/285 (70.9) | 9/22 (40.9) | 15/15 (100) | 56/90 (62.2) | 401/576 (69.6) |
| HCW of unknown category | 17 | 47 | 9 | 8 | 15 | 96 |

**Table S3, footnote.**

Response rates are expressed as numbers of responses/total numbers of eligible healthcare workers (%). HCW=healthcare workers.
