# Supplementary material for: Personal Decision-Making Criteria Related to Seasonal and Pandemic A(H1N1) Influenza-Vaccination Acceptance among French Healthcare Workers
Source: PLoS One. 2012 Jul 27;7(7):e38646. doi: 10.1371/journal.pone.0038646 (PMC3407215; doi:10.1371/journal.pone.0038646)
Supplement: Appendix S1 — English version of the questionnaire used in the INFLUENCE A study. (DOC) [file pone.0038646.s014.doc]

**Supporting information**

**APPENDIX 1.** Self-administered questionnaire

**During the 3 winters preceding the 2009 PANDEMIC A(H1N1) flu, did you get vaccinated against SEASONAL flu?** Never □ Once □ Twice □ Every year □

**Last winter, did you get vaccinated against SEASONAL flu? Yes □No □**

If so, by whom? Occupational Medicine staff □General practitioner □Other □

**Last winter, did you get vaccinated against PANDEMIC A(H1N1) flu? Yes □ No □**

If so, by whom? Occupational medicine staff □Vaccination Center staff □Other □

**Last winter, did your FAMILY CIRCLE have one or more case(s) of:**

**Mild flu or suspected flu (no hospitalization required)?Yes □No □**

If so, was it: SEASONAL flu? □PANDEMIC A(H1N1) flu? □I don’t know □

**Severe flu or suspected flu (hospitalization required)? Yes □No □**

If so, was it: SEASONAL flu? □PANDEMIC A(H1N1) flu? □I don’t know □

**Flu-related death(s)?Yes □No □**

If so, was it: SEASONAL flu? □PANDEMIC A(H1N1) flu? □I don’t know □

**Last winter, did you have among YOUR PATIENTS one or more case(s) of:**

**Flu?Yes □** **No □**

If so, was it: SEASONAL flu? □PANDEMIC A(H1N1) flu? □I don’t know □

**Flu-related death(s)?Yes □** **No □**

If so, was it: SEASONAL flu? □PANDEMIC A(H1N1) flu? □I don’t know □

**Last winter, did you experience flu symptoms? Yes □ No □**

If so, please answer the following questions:

**Was it**: SEASONAL flu? □PANDEMIC A(H1N1) flu? □I don’t know □

**Were you confined to bed because of these flu symptoms?**

Yes □ No □ If so, total number of days in bed: 

**Did you have to consult a doctor for these flu symptoms?**

Yes □ No □ If so, total number of visits: 

**Did you have a TAMIFLU prescription for these flu symptoms?**

Yes □ No □ If so, total number of days on Tamiflu: 

**Did you have to stop working temporarily because of these flu symptoms?**

Yes □ No □ If so, total number of days absent: 

**Did you have to be hospitalized for these flu symptoms?**

Yes □ No □ If so, total number of hospitalization days: 

**Did flu temporarily exacerbate a preexisting chronic disease*? Yes □ No □**

* *Chronic disease: cardiac disease, stroke, cancer, respiratory disease, diabetes mellitus, immunodeficiency.*

**1**

**2**

**3**

**INDIVIDUAL AND ANONYMOUS QUESTIONNAIRE**

**Approximate time required: 10–15 min**

**ATTENTION: questionnaire must be completed fully (incomplete questionnaires will not be retained for analysis)**

| **During the SEASONAL flu-vaccination campaign, to make my decision to be**  **vaccinated or not:** | I strongly agree  **5** | **I agree**  **4** | **I neither agree nor disagree**  **3** | **I disagree**  **2** | **I strongly disagree**  **1** |
| --- | --- | --- | --- | --- | --- |
| I thought that I was **personally** at risk for contracting SEASONAL flu | □ | □ | □ | □ | □ |
| I thought that **my patients** were at risk for contracting SEASONAL flu | □ | □ | □ | □ | □ |
| I thought that **my family circle** was at risk for contracting SEASONAL flu | □ | □ | □ | □ | □ |
| I thought that I could **transmit** SEASONAL flu **to my patients** | □ | □ | □ | □ | □ |
| I thought that I could **transmit** SEASONAL flu **to my family circle** | □ | □ | □ | □ | □ |
| I thought that I was **personally** at risk for **severe** SEASONAL flu | □ | □ | □ | □ | □ |
| I thought that **my patients** were at risk for **severe** SEASONAL flu | □ | □ | □ | □ | □ |
| I thought that **my family circle** was at risk for **severe** SEASONAL flu | □ | □ | □ | □ | □ |
| I thought that **severe cases** of SEASONAL flu would occur **in France** | □ | □ | □ | □ | □ |
| I thought that SEASONAL flu might **prevent me from working** | □ | □ | □ | □ | □ |
| I thought that SEASONAL flu vaccination would **protect me** from contracting SEASONAL flu | □ | □ | □ | □ | □ |
| I thought that healthcare workers’ vaccination against SEASONAL flu would **protect patients** from contracting SEASONAL flu | □ | □ | □ | □ | □ |
| I thought that by getting vaccinated against SEASONAL flu, I would **protect my family circle** from contracting SEASONAL flu | □ | □ | □ | □ | □ |
| I thought that SEASONAL flu vaccination would help **limit its spread** | □ | □ | □ | □ | □ |
| I thought that those who tolerated flu vaccine well last year would **tolerate SEASONAL flu vaccine well this year** | □ | □ | □ | □ | □ |
| I thought that SEASONAL flu vaccination was associated with **frequent side effects** | □ | □ | □ | □ | □ |
| I thought that SEASONAL flu vaccination was associated with **severe side effects** | □ | □ | □ | □ | □ |
| I thought that SEASONAL flu vaccination might **transmit seasonal flu** | □ | □ | □ | □ | □ |
| I thought that the SEASONAL flu vaccination campaign was **not well organized** in my hospital | □ | □ | □ | □ | □ |
| I thought that my **knowledge of SEASONAL flu** was good | □ | □ | □ | □ | □ |
| I thought that my **knowledge of SEASONAL flu vaccination** was good | □ | □ | □ | □ | □ |
| I thought that **paramedical healthcare workers** would get vaccinated against SEASONAL flu | □ | □ | □ | □ | □ |
| I thought that **medical healthcare workers** would get vaccinated against SEASONAL flu | □ | □ | □ | □ | □ |
| I thought that concerning SEASONAL flu vaccination, I had to be **a model for other healthcare workers** | □ | □ | □ | □ | □ |
| I thought that getting myself vaccinated against SEASONAL flu would satisfy **my patients’ expectations** | □ | □ | □ | □ | □ |
| I thought that getting myself vaccinated against SEASONAL flu would satisfy **my colleagues’ expectations** | □ | □ | □ | □ | □ |
| I thought that getting myself vaccinated against SEASONAL flu would satisfy **my family circle’s expectations** | □ | □ | □ | □ | □ |
| I thought that having been vaccinated against seasonal flu **in past years** would protect against  **this year’s** SEASONAL flu | □ | □ | □ | □ | □ |
| I thought that SEASONAL flu vaccination would **preserve my health** | □ | □ | □ | □ | □ |
| I thought that concerning SEASONAL flu vaccination, I had to trust the guidelines of **health authorities** | □ | □ | □ | □ | □ |
| I thought that concerning SEASONAL flu vaccination, I had to consider the information provided by the **media** | □ | □ | □ | □ | □ |
| I thought that concerning SEASONAL flu vaccination, I had to follow the guidelines established in **my ward** | □ | □ | □ | □ | □ |
| I thought that concerning SEASONAL flu vaccination, I had to follow the advice of **my general practitioner** | □ | □ | □ | □ | □ |
| I thought that the **benefit** of SEASONAL flu vaccination was greater than its related risks | □ | □ | □ | □ | □ |

**4SEASONAL FLU: S QUESTIONNAIRE**

**5PANDEMIC A(H1N1) FLU: A QUESTIONNAIRE**

| **During the PANDEMIC A(H1N1) flu-vaccination campaign, to make my decision to be**  **vaccinated or not:** | I strongly agree  5 | **I agree**  **4** | **I neither agree nor disagree**  **3** | **I disagree**  **2** | **I strongly disagree**  **1** |
| --- | --- | --- | --- | --- | --- |
| I thought that I was **personally** at risk for contracting PANDEMIC A(H1N1) flu | □ | □ | □ | □ | □ |
| I thought that **my patients** were at risk for contracting PANDEMIC A(H1N1) flu | □ | □ | □ | □ | □ |
| I thought that **my family circle** was at risk for contracting PANDEMIC A(H1N1) flu | □ | □ | □ | □ | □ |
| I thought that I could **transmit** PANDEMIC A(H1N1) flu **to my patients** | □ | □ | □ | □ | □ |
| I thought that I could **transmit** PANDEMIC A(H1N1) flu **to my family circle** | □ | □ | □ | □ | □ |
| I thought that I was **personally** at risk for **severe** PANDEMIC A(H1N1) flu | □ | □ | □ | □ | □ |
| I thought that **my patients** were at risk for **severe** PANDEMIC A(H1N1) flu | □ | □ | □ | □ | □ |
| I thought that **my family circle** was at risk for **severe** PANDEMIC A(H1N1) flu | □ | □ | □ | □ | □ |
| I thought that **severe cases** of PANDEMIC A(H1N1) flu would occur **in France** | □ | □ | □ | □ | □ |
| I thought that PANDEMIC A(H1N1) flu might **prevent me from working** | □ | □ | □ | □ | □ |
| I thought that PANDEMIC A(H1N1) flu vaccination would **protect me** from contracting PANDEMIC flu | □ | □ | □ | □ | □ |
| I thought that healthcare workers’ vaccination against PANDEMIC A(H1N1) flu would **protect the patients** from contracting PANDEMIC flu | □ | □ | □ | □ | □ |
| I thought that by getting vaccinated against PANDEMIC A(H1N1) flu, I would **protect my family circle** from contracting PANDEMIC flu | □ | □ | □ | □ | □ |
| I thought that PANDEMIC A(H1N1) flu vaccination would help **limit its spread** | □ | □ | □ | □ | □ |
| I thought that those who tolerated flu vaccine well last year would **tolerate** PANDEMIC **A(H1N1) flu vaccine well this year** | □ | □ | □ | □ | □ |
| I thought that PANDEMIC A(H1N1) flu vaccination was associated with **frequent side effects** | □ | □ | □ | □ | □ |
| I thought that PANDEMIC A(H1N1) flu vaccination was associated with **severe side effects** | □ | □ | □ | □ | □ |
| I thought that PANDEMIC A(H1N1) flu vaccination might **transmit A(H1N1) flu** | □ | □ | □ | □ | □ |
| I thought that the PANDEMIC A(H1N1) flu vaccination campaign was **not well organized** in my hospital | □ | □ | □ | □ | □ |
| I thought that my **knowledge of PANDEMIC A(H1N1) flu** was good | □ | □ | □ | □ | □ |
| I thought that my **knowledge of** PANDEMIC **A(H1N1) flu vaccination** was good | □ | □ | □ | □ | □ |
| I thought that **paramedical healthcare workers** would get vaccinated against PANDEMIC A(H1N1) flu | □ | □ | □ | □ | □ |
| I thought that **medical healthcare workers** would get vaccinated against PANDEMIC A(H1N1) flu | □ | □ | □ | □ | □ |
| I thought that concerning PANDEMIC A(H1N1) flu vaccination, I had to be **a model for other healthcare workers** | □ | □ | □ | □ | □ |
| I thought that getting myself vaccinated against PANDEMIC A(H1N1) flu would satisfy **my patients’ expectations** | □ | □ | □ | □ | □ |
| I thought that getting myself vaccinated against PANDEMIC A(H1N1) flu would satisfy **my colleagues’ expectations** | □ | □ | □ | □ | □ |
| I thought that getting myself vaccinated against PANDEMIC A(H1N1) flu would satisfy **my family circle’s expectations** | □ | □ | □ | □ | □ |
| I thought that having been vaccinated against seasonal flu **in past years** would protect against **this year’s**  PANDEMIC A(H1N1) flu | □ | □ | □ | □ | □ |
| I thought that PANDEMIC A(H1N1) flu vaccination would **preserve my health** | □ | □ | □ | □ | □ |
| I thought that concerning PANDEMIC A (H1N1) flu vaccination, I had to trust the guidelines of **health authorities** | □ | □ | □ | □ | □ |
| I thought that concerning PANDEMIC A(H1N1) flu vaccination, I had to consider the information provided by the **media** | □ | □ | □ | □ | □ |
| I thought that concerning PANDEMIC A(H1N1) flu vaccination, I had to follow the guidelines established in **my ward** | □ | □ | □ | □ | □ |
| I thought that concerning PANDEMIC A(H1N1) flu vaccination, I had to follow the advice of **my general practitioner** | □ | □ | □ | □ | □ |
| I thought that the **benefit** of PANDEMIC A(H1N1) flu vaccination was greater than its related risks | □ | □ | □ | □ | □ |

**You are:** A woman □ A man □ **Your age:** years old

**Your job:**

Nurse’s aide □ Nurse □ Head nurse □ Paramedical student □ Midwife □ Orderly □

Medical student □ Resident □ Senior physician □ Physiotherapist □

Other (specify) □ /________________/

**Your type of ward:**

Emergency department □ Intensive care unit □ Medical (acute) □

Rehabilitation/Long-term care □ Psychiatry □ Obstetrics □

Surgery □ Other (specify) □ /_____________/

**Your population of patients:** Adult □ Pediatric □

**Your working hours** (excluding “on-call” hours)**:** Day □ Night □ Day/Night rotation □

**Did you live alone during the 2009–2010 winter?**  **Yes □ No □**

If NO, please answer to the following questions:

Did you live with at least one child under age 6? Yes □ No □

Did you live with a pregnant woman? Yes □ No □

Did you live with at least one person over 65 years old? Yes □ No □

Did you live with at least one person suffering from a chronic disease*? Yes □ No □

Total number of persons living in your home (including you): 

**Were you pregnant during the 2009–2010 winter? Yes □ No □**

**Are you suffering from a chronic disease*? Yes □ No □**

**Chronic disease: cardiac disease, stroke, cancer, respiratory disease, diabetes mellitus, immunodeficiency.*

Considering the information that you have now (media, personal hindsight…), if you had to do it all over again, would you get vaccinated against:

**SEASONAL flu:** Yes □ No □ I don’t know □

**PANDEMIC A(H1N1) flu:** Yes □ No □ I don’t know □

For the coming vaccination campaign, will you get vaccinated against:

**SEASONAL flu?** Yes □ No □ I don’t know yet □

**PANDEMIC A(H1N1) flu?** Yes □ No □ I don’t know yet □

**6**

**7**

**THANK YOU FOR YOUR PARTICIPATION**

**8**
