# Supplementary material for: Personal Decision-Making Criteria Related to Seasonal and Pandemic A(H1N1) Influenza-Vaccination Acceptance among French Healthcare Workers
Source: PLoS One. 2012 Jul 27;7(7):e38646. doi: 10.1371/journal.pone.0038646 (PMC3407215; doi:10.1371/journal.pone.0038646)
Supplement: Appendix S2 — Numbers of missing data per item for the self-administered questionnaire. (DOC) [file pone.0038646.s015.doc]

**Supporting information**

Appendix 2. Numbers of missing data per item for the self-administered questionnaire

***Questionnaire, page 1/4***

| **Item** | **Missing data, n** |
| --- | --- |
| ***Section 2*** | |
| During the 3 winters preceding the 2009 PANDEMIC A(H1N1) flu, did you get vaccinated against seasonal flu? | 73 |
| Last winter, did you get vaccinated against SEASONAL flu? | 53 |
| *If so, by whom?* | 312 |
| Last winter, did you get vaccinated against PANDEMIC A(H1N1) flu? | 54 |
| *If so, by whom?* | 251 |
| ***Section 2*** | |
| Last winter, did your FAMILY CIRCLE have one or more case(s) of mild flu or suspected flu (no hospitalization required)? | 48 |
| *If so, was it SEASONAL flu? /* PANDEMIC *A(H1N1) flu? / I don’t know* | 199 |
| Last winter, did you have in YOUR FAMILY CIRCLE one or more case(s) of severe flu or suspected flu (hospitalization required)? | 47 |
| *If so, was it SEASONAL flu? / PANDEMIC A(H1N1) flu? / I don’t know* | 77 |
| Last winter, did you have in YOUR FAMILY CIRCLE one or more case(s) of flu-related death(s)? | 62 |
| *If so, was it SEASONAL flu? / PANDEMIC A(H1N1) flu? / I don’t know* | 67 |
| Last winter, did you have among YOUR PATIENTS one or more case(s) of flu? | 112 |
| *If so, was it SEASONAL flu? / PANDEMIC A(H1N1) flu? / I don’t know* | 375 |
| Last winter, did you have among YOUR PATIENTS one or more case(s) of flu-related death(s)? | 147 |
| *If so, was it SEASONAL flu? / PANDEMIC A(H1N1) flu? / I don’t know* | 170 |
| ***Section 3*** | |
| Last winter, did you experience flu symptoms? | 32 |
| *If so, was it SEASONAL flu? / PANDEMIC A(H1N1) flu? / I don’t know* | 50 |
| Were you confined to bed because of these flu symptoms? | 245 |
| *If so, total number of days in bed:* | 245 |
| Did you have to consult a doctor for these flu symptoms? | 36 |
| *If so, total number of visits:* | 141 |
| Did you have a Tamiflu prescription for these flu symptoms? | 41 |
| *If so, total number of days on Tamiflu:* | 36 |
| Did you have to stop working temporarily because of these symptoms? | 41 |
| *If so, total number of days absent:* | 94 |
| Did you have to be hospitalized for these flu symptoms? | 38 |
| *If so, total number of hospitalization days:* | 34 |
| Did flu temporarily exacerbate a preexisting chronic disease? | 70 |
| ***Section 4: SEASONAL FLU: S QUESTIONNAIRE*** | |
| 1. I thought that I was personally at risk for contracting SEASONAL flu | 68 |
| 1. I thought that my patients were at risk for contracting SEASONAL flu | 87 |
| 1. I thought that my family circle was at risk for contracting SEASONAL flu | 104 |
| 1. I thought that I could transmit SEASONAL flu to my patients | 100 |
| 1. I thought that I could transmit SEASONAL flu to my family circle | 113 |
| 1. I thought that I was personally at risk for severe SEASONAL flu | 120 |
| 1. I thought that my patients were at risk for severe SEASONAL flu | 130 |
| 1. I thought that my family circle was at risk for severe SEASONAL flu | 152 |
| 1. I thought that severe cases of SEASONAL flu would occur in France | 141 |
| 1. I thought that SEASONAL flu might prevent me from working | 132 |
| 1. I thought that SEASONAL flu vaccination would protect me from contracting SEASONAL flu | 139 |
| 1. I thought that healthcare workers’ vaccination against SEASONAL flu would protect patients from contracting SEASONAL flu | 124 |
| 1. I thought that by getting vaccinated against SEASONAL flu, I would protect my family circle from contracting SEASONAL flu | 134 |
| 1. I thought that SEASONAL flu vaccination would help limit its spread | 125 |
| 1. I thought that those who tolerated flu vaccine well last year would tolerate this vaccine well this year | 135 |
| 1. I thought that SEASONAL flu vaccination was associated with frequent side effects | 118 |
| 1. I thought that SEASONAL flu vaccination was associated with severe side effects | 131 |
| 1. I thought that SEASONAL flu vaccination might transmit SEASONAL flu | 154 |
| 1. I thought that the SEASONAL flu vaccination campaign was not well organized in my hospital | 152 |
| 1. I thought that my knowledge of SEASONAL flu was good | 155 |
| 1. I thought that my knowledge of SEASONAL flu vaccination was good | 149 |
| 1. I thought that paramedical healthcare workers would get vaccinated against SEASONAL flu | 142 |
| 1. I thought that medical healthcare workers would get vaccinated against SEASONAL flu | 135 |
| 1. I thought that concerning SEASONAL flu vaccination, I had to be a model for other healthcare workers | 141 |
| 1. I thought that getting myself vaccinated against SEASONAL flu would satisfy my patients’ expectations | 132 |
| 1. I thought that getting myself vaccinated against SEASONAL flu would satisfy my colleagues’ expectations | 134 |
| 1. I thought that getting myself vaccinated against SEASONAL flu would satisfy my family-circle’s expectations | 134 |
| 1. I thought that getting vaccinated against seasonal flu in past years would protect me against SEASONAL flu this year | 145 |
| 1. I thought that SEASONAL flu vaccination would preserve my health | 129 |
| 1. I thought that concerning SEASONAL flu vaccination, I had to trust the guidelines of health authorities | 145 |
| 1. I thought that concerning SEASONAL flu vaccination, I had to consider the information provided by the media | 137 |
| 1. I thought that concerning SEASONAL flu vaccination, I had to follow the guidelines established in my ward | 145 |
| 1. I thought that concerning SEASONAL flu vaccination, I had to follow the advice of my general practitioner | 134 |
| 1. I thought that the benefit of SEASONAL flu vaccination was greater than its related risks |  |

| **Item** | **Missing data, n** |
| --- | --- |
| ***Section 5 PADEMIC A (H1N1) FLU: A QUESTIONNAIRE*** | |
| 1. I thought that I was personally at risk for contracting PANDEMIC A(H1N1) flu | 109 |
| 1. I thought that my patients were at risk for contracting PANDEMIC A(H1N1) flu | 119 |
| 1. I thought that my family circle was at risk for contracting PANDEMIC A(H1N1) flu | 132 |
| 1. I thought that I could transmit PANDEMIC A(H1N1) flu to my patients | 137 |
| 1. I thought that I could transmit PANDEMIC A(H1N1) flu to my family circle | 143 |
| 1. I thought that I was personally at risk for severe PANDEMIC A(H1N1) flu | 149 |
| 1. I thought that my patients were at risk for severe PANDEMIC A(H1N1) flu | 152 |
| 1. I thought that my family circle was at risk for severe PANDEMIC A(H1N1) flu | 149 |
| 1. I thought that severe cases of PANDEMIC A(H1N1) flu would occur in France | 157 |
| 1. I thought that PANDEMIC A(H1N1) flu might prevent me from working | 153 |
| 1. I thought that PANDEMIC A(H1N1) flu vaccination would protect me from contracting PANDEMIC flu | 155 |
| 1. I thought that healthcare workers’ vaccination against PANDEMIC A(H1N1) flu would protect the patients from contracting PANDEMIC flu | 151 |
| 1. I thought that by getting vaccinated against PANDEMIC A(H1N1) flu, I would protect my family circle from contracting PANDEMIC flu | 159 |
| 1. I thought that A(H1N1) flu vaccination would help limit its spread | 149 |
| 1. I thought that those who tolerated flu vaccine well last year would tolerate PANDEMIC A(H1N1) vaccine well this year | 156 |
| 1. I thought that PANDEMIC A(H1N1) flu vaccination was associated with frequent side effects | 154 |
| 1. I thought that PANDEMIC A(H1N1) flu vaccination was associated with severe side effects | 157 |
| 1. I thought that A(H1N1) flu vaccination might transmit PANDEMIC A(H1N1) flu | 175 |
| 1. I thought that the PANDEMIC A(H1N1) flu vaccination campaign was not well organized in my hospital | 174 |
| 1. I thought that my knowledge of PANDEMIC A(H1N1) flu was good | 173 |
| 1. I thought that my knowledge of PANDEMIC A(H1N1) flu vaccination was good | 167 |
| 1. I thought that paramedical healthcare workers would get vaccinated against PANDEMIC A(H1N1) flu | 166 |
| 1. I thought that medical healthcare workers would get vaccinated against PANDEMIC A(H1N1) flu | 169 |
| 1. I thought that concerning PANDEMIC A(H1N1) flu vaccination, I had to be a model for other healthcare workers | 155 |
| 1. I thought that getting myself vaccinated against PANDEMIC A(H1N1) flu would satisfy my patients’ expectations | 155 |
| 1. I thought that getting myself vaccinated against PANDEMIC A(H1N1) flu would satisfy my colleagues’ expectations | 155 |
| 1. I thought that getting myself vaccinated against PANDEMIC A(H1N1) flu would satisfy my family circle’s expectations | 157 |
| 1. I thought that having been vaccinated against seasonal flu in past years would protect me against this year’s PANDEMIC A(H1N1) flu | 157 |
| 1. I thought that, by getting vaccinated against PANDEMIC A(H1N1) flu, I would preserve my health | 162 |
| 1. I thought that concerning PANDEMIC A(H1N1) flu vaccination, I had to trust the guidelines of health authorities | 160 |
| 1. I thought that concerning PANDEMIC A(H1N1) flu vaccination, I had to consider the information provide by the media | 152 |
| 1. I thought that concerning PANDEMIC A(H1N1) flu vaccination, I had to follow the guidelines established in my ward | 149 |
| 1. I thought that concerning PANDEMIC A(H1N1) flu vaccination, I had to follow the advice of my general practitioner | 140 |
| 1. I thought that the benefit of PANDEMIC A(H1N1) flu vaccination was greater than its related risks |  |
| ***Section 6*** | |
| You are: a woman / a man? | 90 |
| Your age? | 329 |
| Your job? | 96 |
| Your type of ward? | 151 |
| Your population of patients? | 234 |
| Your working hours (excluding “on-call” hours)? | 195 |
| ***Section 7*** | |
| Did you live alone during the 2009–2010 winter? | 161 |
| Did you live with at least one child under age 6? | 298 |
| Did you live with a pregnant woman? | 357 |
| Did you live with at least one person over 65 years old? | 343 |
| Did you live with at least one person suffering from a chronic disease? | 336 |
| Total number of persons living in your home (including you)? | 399 |
| Were you pregnant during the 2009–2010 winter? | 157 |
| Are you suffering from a chronic disease? | 119 |
| ***Section 8*** | |
| Considering the information that you have now (media, personal hindsight…), if you had to do it all over again, would you get vaccinated against SEASONAL flu? | 98 |
| Considering the information that you have now (media, personal hindsight…), if you had to do it all over again, would you get vaccinated against PANDEMIC A(H1N1) flu? | 107 |
| For the coming vaccination campaign, will you get vaccinated against SEASONAL flu? | 98 |
| For the coming vaccination campaign, will you get vaccinated against PANDEMIC A(H1N1) flu? | 108 |
